# Molecular Targeting of the Peripheral Nerve–Tumor Axis in Extracranial Solid Cancers: Eight Signaling Systems, One Effect—Systematic Review and Multilevel Meta-Analysis

**DOI:** 10.3390/ijms27209090

**Published:** 2026-10-14

**Authors:** Diana-Rodica Tudorașcu, Cristin Constantin Vere, Mihai Petrescu, Ana-Maria Ciurea, Răzvan-Cosmin Pană, Alexandra Oltea Dan, Elena-Anca Târtea, Andrei Greșiță, Diana-Ruxandra Hădăreanu, Cosmin Vasile Obleagă, Georgică Târtea

**Affiliations:** 1 Department of Medical Semiology, University of Medicine and Pharmacy of Craiova, 2 Petru Rares Street, 200349 Craiova, Romania; diana.tudorascu@umfcv.ro; 2 Department of Gastroenterology, University of Medicine and Pharmacy of Craiova, 200349 Craiova, Romania; cristin.vere@umfcv.ro; 3 Department of Psychiatry, University of Medicine and Pharmacy of Craiova, 200349 Craiova, Romania; mihai.petrescu@umfcv.ro; 4 Department of Oncology, University of Medicine and Pharmacy of Craiova, 200349 Craiova, Romania; ana.ciurea@umfcv.ro; 5 Department of Gynecology, University of Medicine and Pharmacy of Craiova, 2 Petru Rares Street, 200349 Craiova, Romania; razvan.pana@umfcv.ro; 6 Experimental Research Centre for Normal and Pathological Aging, University of Medicine and Pharmacy of Craiova, 2 Petru Rares Street, 200349 Craiova, Romania; alexandra.dan@umfcv.ro (A.O.D.); andrei.gresita@umfcv.ro (A.G.); georgica.tartea@umfcv.ro (G.T.); 7 Department of Neurology, University of Medicine and Pharmacy of Craiova, 2 Petru Rares Street, 200349 Craiova, Romania; 8 Department of Cardiology, University of Medicine and Pharmacy of Craiova, 2 Petru Rares Street, 200349 Craiova, Romania; diana.hadareanu@umfcv.ro; 9 Department of Surgical Oncology, University of Medicine and Pharmacy of Craiova, 200349 Craiova, Romania; cosmin.obleaga@umfcv.ro

**Keywords:** tumor innervation, adrenergic receptors, muscarinic receptors, neurotrophins, axon guidance molecules, perineural invasion, cancer neuroscience

## Abstract

The peripheral nerves that innervate solid tumors communicate with the malignant cells through a restricted set of molecular signaling systems: adrenergic and muscarinic receptors, sensory neuropeptides, neurotrophins and their tyrosine kinase receptors, axon guidance molecules, and nociceptor ion channels. Each has been proposed separately as a therapeutic target but never quantitatively compared with the others. The question is whether one of them dominates—or whether the real target is the axis as a system. We systematically searched PubMed, Scopus, and Web of Science, with four query modules per database, without year or language restriction. We included in vivo studies in mammals, on extracranial solid cancers, with an intervention that modulated tumor innervation, peripheral nerve activity, or neural signaling, and with at least one tumor burden outcome. Of 16,649 unique records, 362 were included, with 1291 experiments and 1437 effects. Blockade of the peripheral nerve–tumor axis reduced tumor burden by −44.8%. In the prespecified primary analysis, on established tumors, the reduction was −52.0% with no significant difference from preventive interventions (*p* = 0.17). All eight molecular signaling systems responded to blockade, with estimates ranging from −34.3% for TRP channels to −70.2% for glutamatergic signaling. Interventions that activate the axis produced the opposite effect, with a 52.1% increase (95% CI: 32.1 to 75.1) across 253 effects, and bidirectionality was demonstrated within the same molecular system for the adrenergic compartment (−42.8% for blockade vs. 58.1% for activation), axon guidance molecules, and TRP channels. Egger’s test indicated marked small-study effects (slope −1.12; *p* < 0.001); the estimate remained stable when low-confidence digitized values were excluded (−42.6%). Eight distinct molecular signaling systems, disrupted by methods with nothing in common, produce reductions of a similar magnitude in tumor burden, and stimulation of three of them produces the opposite effect.

## 1. Introduction

The idea that solid tumors build their own innervation and that this innervation serves their growth has moved from histological observation to a therapeutic program in the last fifteen years. Intratumoral nerve fiber density correlates with aggressiveness and survival in prostate, pancreatic, stomach, and colorectal cancers [1], and tumor axonogenesis is an active process, dependent on neurotrophins secreted by malignant cells and stromal cells [1,2]. The mechanisms described so far cover all three compartments of the peripheral nervous system. Sympathetic fibers, through β-adrenergic receptors, promote angiogenic switching and immunosuppressive remodeling of the microenvironment [3,4,5]. Parasympathetic fibers, through muscarinic receptors, support the expansion of tumor stem cells in the stomach and colon [6,7,8]. Sensory fibers release neuropeptides—calcitonin gene-related peptide (CGRP), substance P, and vasoactive intestinal peptide (VIP)—that modulate both proliferation and lymphocyte infiltration [9,10,11,12,13]. Perineural invasion, long considered a prognostic marker, has been shown to be a bidirectional interaction, in which tumor cells and Schwann cells reprogram each other [14,15,16,17]. The clinical correlates of this axis have also been documented in human cohorts. In colorectal cancer, nerve fiber density and synaptophysin expression are associated with survival [18,19], and the expression of the sensory peptides—substance P and CGRP, along with their calcitonin receptor-like receptor (CRLR)—varies systematically with tumor stage [20,21]. In gastric cancer, tissue concentrations of adrenaline and noradrenaline and markers of sympathetic activation are independent negative prognostic factors [22,23], and β2-adrenergic receptors are differentially expressed in the enteric nerve plexuses adjacent to the tumor [24]. Heart rate variability at diagnosis, as an integrative measure of autonomic tone, correlates with survival in colorectal cancer [25], suggesting that the nervous influence on tumor progression is measurable at a systemic level, not just locally [26].

However, the preclinical evidence has been uneven. Most studies apply neural intervention before tumor inoculation or immediately after, i.e., in a window of prevention that does not correspond to the clinical situation, in which the patient presents with an already established tumor. A second reason for caution is the direction of intervention: some of the literature increases neural signaling—through chronic stress, exogenous catecholamines, or agonists—and it is not clear that these studies can be treated as mirror images of blockade. Finally, the effect sizes come from very different models, species, and outcomes, making it difficult for a narrative synthesis to distinguish signals from noise.

Quantitative syntheses in the field are few and limited to either a single class of interventions or a single tumor type. No meta-analysis has yet addressed the peripheral nerve–tumor axis as a whole, with an explicit taxonomy of the level of intervention and the timing of its application, and none has used a model that respects the real hierarchical structure of preclinical data, where one article contributes multiple experiments and one experiment multiple effects [27,28,29,30].

This systematic review and multilevel meta-analysis aims to answer four questions. First: Does blocking the peripheral nerve–tumor axis reduce tumor burden in in vivo models of extracranial solid cancers? Second: Is the effect maintained when the intervention is applied to an already established tumor, i.e., within the clinically relevant therapeutic window? Third: Does the effect depend on the targeted fiber, the molecular level of the intervention, or the tumor type? Fourth: Do interventions that increase neural signaling produce the inversely symmetrical effect?

**Figure 1:**
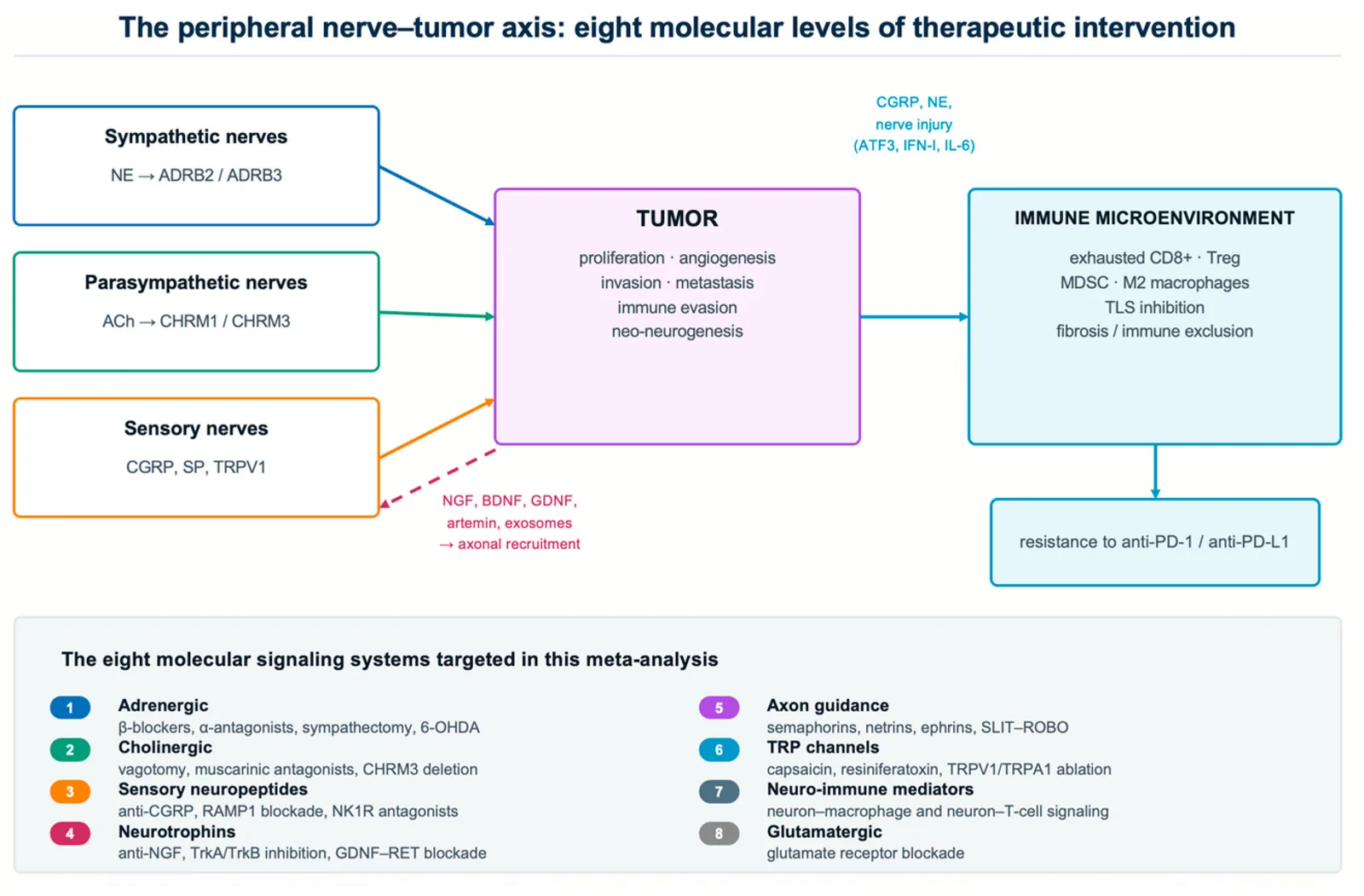
The peripheral nerve–tumor axis and the eight molecular signaling systems targeted in this meta-analysis. Sympathetic, parasympathetic and sensory fibers innervate solid tumors and act on the malignant cell through distinct receptor families: sympathetic terminals release norepinephrine (NE) onto β2- and β3-adrenergic receptors (ADRB2, ADRB3); parasympathetic terminals release acetylcholine (ACh) onto M1 and M3 muscarinic receptors (CHRM1, CHRM3); and sensory nociceptors release calcitonin gene-related peptide (CGRP) and substance P (SP) and signal through transient receptor potential vanilloid 1 (TRPV1). Neural input promotes proliferation, angiogenesis, invasion, metastasis, immune evasion and further axonal ingrowth (neo-neurogenesis). The tumor in turn recruits axons by secreting the neurotrophins nerve growth factor (NGF), brain-derived neurotrophic factor (BDNF), glial cell-line-derived neurotrophic factor (GDNF) and artemin, and by releasing exosomes (dashed arrow), establishing a self-reinforcing loop. Signals passed from the innervated tumor to the immune compartment—CGRP, NE and mediators of nerve injury, including activating transcription factor 3 (ATF3), type I interferon (IFN-I) and interleukin-6 (IL-6)—shape an immunosuppressive microenvironment characterized by exhausted CD8-positive T cells, regulatory T cells (Treg), myeloid-derived suppressor cells (MDSC) and M2-polarized macrophages, with inhibition of tertiary lymphoid structures (TLSs) and fibrotic immune exclusion, which contributes to resistance to anti-programmed cell death protein 1 (anti-PD-1) and anti-programmed death-ligand 1 (anti-PD-L1) therapy. The lower panel lists the eight molecular signaling systems into which every experiment was classified post hoc, independently of the method used, together with representative interventions applied to interrupt each system in the included studies.

The conceptual model of the peripheral nerve–tumor axis, with the signaling systems involved and the points at which each was disrupted or stimulated in the included studies, is shown in Figure 1. It serves as a reading frame for the analysis that follows, in which the eight systems are quantified in turn.

## 2. Materials and Methods

### 2.1. Protocol and Registration

The review was conducted according to the PRISMA 2020 (Preferred Reporting Items for Systematic Reviews and Meta-Analyses) statement [31] and reported according to the PRISMA-S (PRISMA for Searching) extension for search strategies [32]. The protocol was drafted according to the PRISMA-P (PRISMA for Protocols) recommendations [33] and structured for registration in PROSPERO4animals (International Prospective Register of Systematic Reviews—extension for animal studies), the registry dedicated to preclinical syntheses [34]. Because the PROSPERO4animals guidance does not accept the registration of reviews in which data extraction has already begun, the protocol was deposited with the Open Science Framework as a time-stamped registration with a persistent identifier: https://doi.org/10.17605/OSF.IO/DZH4J (deposited on 31 August 2026). The deposit is retrospective with respect to the conduct of the review and is explicitly declared as such in the registration: the document deposited is the protocol drafted before data extraction, together with the seven amendments adopted after extraction and before any statistical analysis. Nothing in the deposited protocol was revised in the light of the results. Deviations from the protocol as originally written—three databases interrogated instead of the five planned, a fourth query module added to the three foreseen, the absence of manual reference-list checking, and assessment of screening reliability for 240 rather than 200 records—are declared in full in the registration; none was adopted in response to observed results.

The protocol was amended in seven points, after the completion of data extraction and before any statistical analysis. The timing of the amendment is explicitly stated, and the amendments are reported in full in Supplementary Material S1. They concern: the extension of the primary outcome hierarchy by a fifth level (extent of perineural invasion measured continuously); maintaining the exclusion of pulmonary retention models after intravenous injection; conditional inclusion of interventions on central neurons, only with a demonstrated peripheral sympathetic link; exclusion in principle of tumors of the nerve itself; separate analysis of axis activation studies; the multiple report deduplication rule; and continuity correction for zero-mean effects.

### 2.2. Eligibility Criteria

Screening was managed with tools dedicated to systematic syntheses [35], and methodological filters for identifying animal studies were adapted from the specialized literature [36,37]. The general methodology of the preclinical study syntheses followed the established recommendations [38,39].

We included studies that simultaneously fulfilled ten conditions: in vivo experiment in mammals; extracranial solid tumor; an intervention on tumor innervation, peripheral nerve activity, or neural signaling; justification of the intervention as targeting the nerve–tumor axis, or measurement of innervation, neuronal activity, or local neurotransmitter concentration; concurrent control group; at least one tumor burden outcome; other than an NTRK inhibitor administered for an oncogenic fusion; objective other than exclusively analgesia or neuroprotection; original study; and sufficient reporting for extraction.

Primary outcome hierarchy, in order of preference: tumor mass at sacrifice; tumor volume at the last time point common to all groups; histological tumor area; bioluminescent or imaging signal; and—by amendment 1—extent of perineural invasion measured continuously, as length of invasion along the nerve, volume, diameter, or area of the invaded nerve, or numerical severity score. Binary incidence of perineural invasion, sciatic nerve function score, intratumoral innervation density, and number of invaded nerves per field were not accepted as outcomes.

### 2.3. Information Sources and Search Strategy

We queried PubMed, Scopus, and Web of Science, each with four complementary search modules: a main module focused on tumor innervation, a module on neurotransmitters and receptors, a module on neurotrophins and axon guidance molecules, and a safety-net module built after testing with benchmark articles. We did not impose restrictions on year, language, or publication type at the query level. The syntax of each query, with the run date and number of records returned, is given in Supplementary Material S2.

The strategy was validated by testing with known articles: a set of benchmark papers in the field was checked for retrieval, and the modules were revised until the only missing benchmark article remained. The overlap between the databases was measured: Web of Science added 2655 new records out of 9725 returned, i.e., an overlap of 73%, which constitutes a quantitative argument for the saturation of the search.

### 2.4. Study Selection

Records were deduplicated by normalized digital object identifier (DOI), then by normalized title with a one-year tolerance for the year of publication. We then applied a relevance filter that retained any record containing an oncology term in the title or abstract, or a phrase specific to the nerve–tumor axis, as well as all records without an abstract. The filter was validated on a set of 113 references known to be relevant: none were lost.

**Figure 2:**
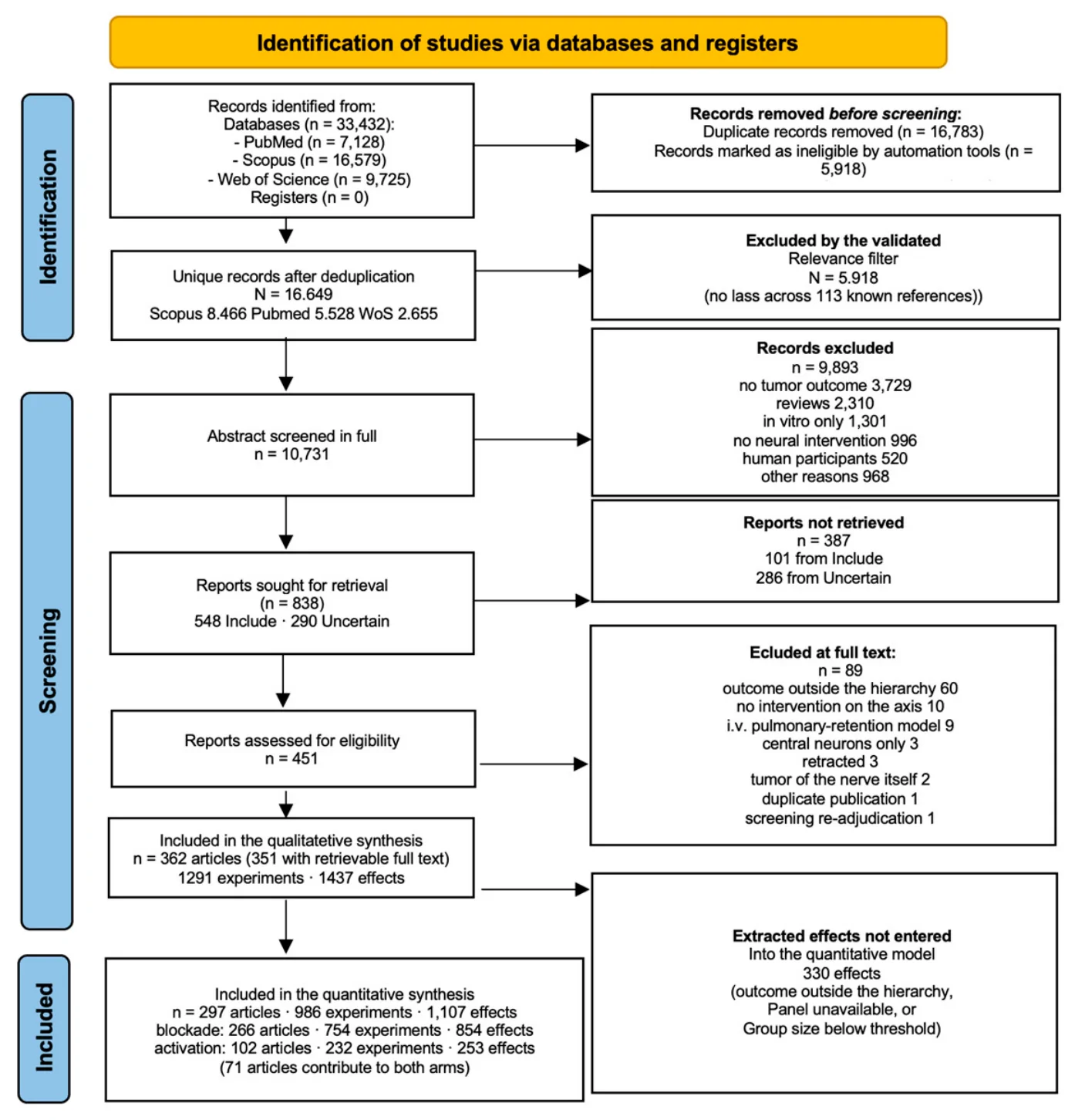
PRISMA 2020 flow diagram. Numbers for each box, together with the full breakdown of exclusion reasons, are given in Supplementary Table S7.

Screening on title and abstract was done by applying the ten eligibility questions, stopping at the first negative answer and recording the reason. Reliability was estimated by independent re-screening of a random sample of 240 records, stratified by decision category (80 Include, 80 Uncertain, 80 Exclude). The gross agreement was 69.2% (Cohen’s kappa 0.54), and the agreement on the retained-versus-excluded decision was 75.0%. None of the 80 exclusions checked were reclassified as eligible. Discordant records were adjudicated by two authors. The complete selection process, with the number of records retained and excluded at each stage, is summarized in the PRISMA 2020 flow diagram (Figure 2).

### 2.5. Data Extraction

Each article was divided into experiments, defined as a unique combination of tumor model × intervention, and each experiment into effects, defined as an intervention-versus-control comparison for the primary outcome. At multiple doses, each dose generated an effect, with the control being marked as common. At multiple measurement times, we retained a single effect, at the last time point common to all groups.

For each effect we extracted, per group, the mean, the measure of dispersion, and the number of animals. The order of sources was: text, tables, supplementary material, figures. Where the article reported the standard error of the mean (SEM), the conversion to the standard deviation (SD) was done using the equation SD = SEM × √n. Where the supplementary material contained individual values for each animal, the mean and standard deviation were recalculated from the raw data, and the mean was the exact result.

Digitization of graphic panels. For effects whose values were only available in the figures, extraction was done in a separate step, after the textual extraction was completed, on the 200 dpi rendered pages of the original article, with cropping to 600 dpi where the error bar was not legible at the page resolution. For each panel, the extreme values and ordinate axis graduations and the scale type—linear, logarithmic, or dashed—were first identified, and the values were obtained by interpolation corresponding to the scale type. The mean was read as the bar height or the position of the mean marker, and the dispersion as the distance from the mean to the upper end of the error bar. For panels without error bars but with individual points visible, the standard deviation was estimated from the scatter of the points. Each value was given a three-level confidence score at the time of reading—high (clear axis, clear bar ends), medium (interpolation between sparse gradations, broken scale, partially obscured bar ends), and low (estimated dispersion from points, logarithmic axis without intermediate gradations, crowded panel)—and the score was subsequently used in a prespecified sensitivity analysis. When the unit in the figure differed from that recorded in the extraction sheet, the value was converted or, if not convertible, reported in the unit of the figure, which does not affect the ratio of means.

Imputation of dispersion. For experimental arms for which neither the figure nor the text provided any measure of dispersion nor individual points, the standard deviation was imputed from the median coefficient of variation of effects at the same level of the outcome hierarchy. Effects containing at least one arm with imputed dispersion were flagged, and a prespecified sensitivity analysis was run with those effects excluded.

Where an article reported medians and quartiles instead of means, conversion was done with validated estimators for the respective sample size [40,41,42].

For each experiment, we coded: the nerve fiber targeted; the level of intervention; the time of application relative to tumor stage (T0 before inoculation, T1 after inoculation but before measurable tumor, T2 after established tumor, T3 after documented progression); neural target engagement (confirmed, probable, unconfirmed); host immune status; model type; route of administration; and direction of intervention (blockade or activation).

#### Note on the Team’s Methodological Experience

The extraction sheet and the coding rules for the quantified parameters were built based on the team’s methodological experience in the evaluation of tissue and systemic markers in other pathological contexts: immunohistochemical quantification of lymphocyte subpopulations and vascular endothelial growth factor A (VEGF-A) in acute pancreatitis [43], the role of skin and muscle biopsy in the diagnosis of connective tissue diseases [44], the evaluation of retinal microvascular parameters by optical coherence tomography (OCT) angiography as a marker of neurovascular damage [45], as well as the electrocardiographic and echocardiographic characterization of cardiac phenotypes in silicosis [46], in left ventricular noncompaction [47] and in infective endocarditis [48]. These papers do not address the nerve–tumor axis and do not contribute data to the synthesis; they are cited exclusively for the procedures for quantifying and evaluating autonomic and cardiovascular parameters that we reused in the construction of the extraction sheet.

### 2.6. Risk of Bias

The risk of bias was assessed using the SYRCLE tool, on its ten domains [49], and the quality of reporting was assessed through the ARRIVE 2.0 (Animal Research: Reporting of In Vivo Experiments) recommendations [50,51]. The assessment of the level of confidence in the evidence followed the preclinical adaptation of the GRADE (Grading of Recommendations, Assessment, Development and Evaluation) approach [52,53].

### 2.7. Effect Size and Statistical Model

The effect size was the logarithmic ratio of the means, lnRR = ln(intervention_mean/control_mean), with variance:

$$\mathrm{Var}\left(\ln\mathrm{RR}\right)=\frac{s_{\mathrm{int}}^{2}}{n_{\mathrm{int}}\;{\bar{x}}_{\mathrm{int}}^{2}}+\frac{s_{\mathrm{ctrl}}^{2}}{n_{\mathrm{ctrl}}\;{\bar{x}}_{\mathrm{ctrl}}^{2}}$$

The choice and implementation of lnRR follows the method proposed for meta-analysis of response reports [54].

Results are expressed as percent change in tumor burden, 100 × (elnRR − 1). The choice of lnRR instead of the standardized mean difference is motivated by the fact that outcomes are strictly positive ratio measures, measured in different units across studies, and lnRR is unit-invariant and directly clinically interpretable.

The synthesis was done using a three-level random-effects model: effects are nested within experiments, and experiments within articles. The model was estimated by restricted maximum likelihood (REML) in Stata 19, with the command meta meregress || art: || expr:. This structure is necessary because an article typically contributes multiple experiments, and an experiment multiple effects; ignoring nesting would underestimate the standard errors [27,28]. Heterogeneity was quantified and decomposed across the two levels [55,56] and reported as total and component I^2^; interpretation of random-effects estimates and the need for prediction intervals followed established recommendations [57,58].

As a robustness check, we also ran a robust variance estimate, which does not assume a particular correlation structure between effects of the same article [59,60,61]. Reuse of control groups in multiple dose comparisons was handled by explicitly marking the common control [29,30].

The prespecified primary analysis included only effects with T2 or T3 and outcomes from levels 1–5 of the hierarchy, from the blockade studies. Axis activation studies were analyzed separately, without sign reversal, according to amendment 5.

Classification by molecular signaling systems (post hoc). The prespecified taxonomy encodes the method by which the axis is disrupted, not the molecular system engaged: a chemical sympathectomy and a β-blocker enter at different levels, although they reach the same noradrenergic pathway. After the prespecified analyses were completed, each experiment was therefore reclassified, independently of method, according to the signaling system that the intervention interrupts or the activation of adrenergic, cholinergic, or sensory neuropeptides; neurotrophins and Trk receptors; axon guidance molecules; nociceptor TRP channels; neuroimmune mediators; or glutamatergic and mixed unspecified agents, for interventions in which the nerve is manipulated without a defined molecular target. Each experiment received a single label, assigned according to a pre-established order of priority, and when the article explicitly stated the target, that was retained. The classification was made on the full text of the intervention description, without access to the effect size. Results across systems are reported as exploratory analyses, distinct from prespecified analyses, and *p* values are not corrected for multiplicity.

Moderation was tested by meta-regression in the same three-level model for: time of intervention, nerve fiber, intervention level, and target engagement.

Small-study effects were assessed by Egger’s test [62], adapted as a meta-regression on the standard error within the three-level model, and visually by funnel plots with significance contours, interpreted according to recommendations for asymmetry [63,64,65]. Publication bias and low statistical power are well-documented problems in the preclinical literature [66,67]. Robustness was tested by successive exclusion of each article.

For effects with a zero mean in one arm, where lnRR is not defined, the continuity correction prespecified by Amendment 7 was applied: adding to both arms half of the smallest non-zero value observed in that experiment, with sensitivity analysis without the corrected effects. In the final base, no effects required this correction, as all control and intervention means were strictly positive.

The data, the full Stata code, and the digitization files are available as Supplementary Materials.

### 2.8. Use of Generative Artificial Intelligence Tools

In carrying out this synthesis, we used an assistant based on a large-scale linguistic model (Claude, Anthropic; Claude Opus 5 version, accessed through the Claude application, 2026) in the following stages, declared here in full:

(i) Deduplication and screening of records—normalization of identifiers and titles, construction of the screening database and application of eligibility criteria in two independent passes, disagreements being adjudicated by the authors;
(ii) Data extraction—transfer of values from the text, tables and Supplementary Materials into the structured extraction sheet;
(iii) Digitization of graphic panels, for effects whose numerical values were not reported individually in the text or tables: identification of the ordinate axis gradations and the scale type, then interpolation of the mean and dispersion, according to the procedure described in Section 2.5;
(iv) Post hoc classification by molecular signaling systems, performed on the intervention description, without access to the effect size, and subsequently verified by the author;
(v) Writing the analysis code in Stata and numerically verifying it by an independent implementation;
(vi) Drafting, linguistic editing, and translation of the text, including the English translation of figures, tables, and Supplementary Materials.

No results were generated by the model: all estimates come from running the code on the extracted dataset, provided in full in the Supplementary Materials. Each digitized value was assigned a three-step confidence score at the time of reading, and the robustness of these values was tested by two sensitivity analyses—exclusion of values with low confidence and imputed dispersion (−42.6%, 764 effects) and restriction to values reported directly in the text or tables (−38.5%, 125 effects)—both consistent with the main estimate of −44.8%. The authors reviewed and edited the entire output and assume full responsibility for the content of this publication.

## 3. Results

### 3.1. Study Selection

The three databases returned, through the four search modules, a total that after deduplication left 16,649 unique records (Scopus 8466, PubMed 5528, and Web of Science 2655). The validated relevance filter eliminated 5918 records, leaving 10,731 abstracts for evaluation. Screening excluded 9893 records; the dominant reasons were the absence of a tumor outcome (3729), the type of publication—syntheses and review articles (2310)—and exclusively in vitro studies (1301).

Thus, we had 838 articles to search in full text, of which 387 could not be obtained. The 451 articles obtained were assessed for eligibility; 89 were excluded from full-text analysis: 60 because they did not report any outcome from the prespecified hierarchy, 10 because they did not contain any intervention that directly modulated tumor innervation or peripheral neural signaling, nine for the pulmonary retention model after intravenous injection, three for intervention on central neurons without demonstrated peripheral sympathetic link, one as a duplicate publication of an already included study, one excluded at screening re-adjudication, two because the tumor studied was a tumor of the nerve itself, and three because they had been withdrawn. The reason for each exclusion is reported individually in Supplementary Material S7. A total of 362 eligible articles remained. For 11 of them, the full text could not be obtained even after evaluation—the available file contained no more than the abstract—so eligibility was established based on the abstract, but no effect could be extracted. The 351 articles actually extracted provided 1291 experiments, of which 1270 had at least one usable effect, and 1437 effects in total (Figure 2).

### 3.2. Characteristics of Included Studies

**Figure 3:**
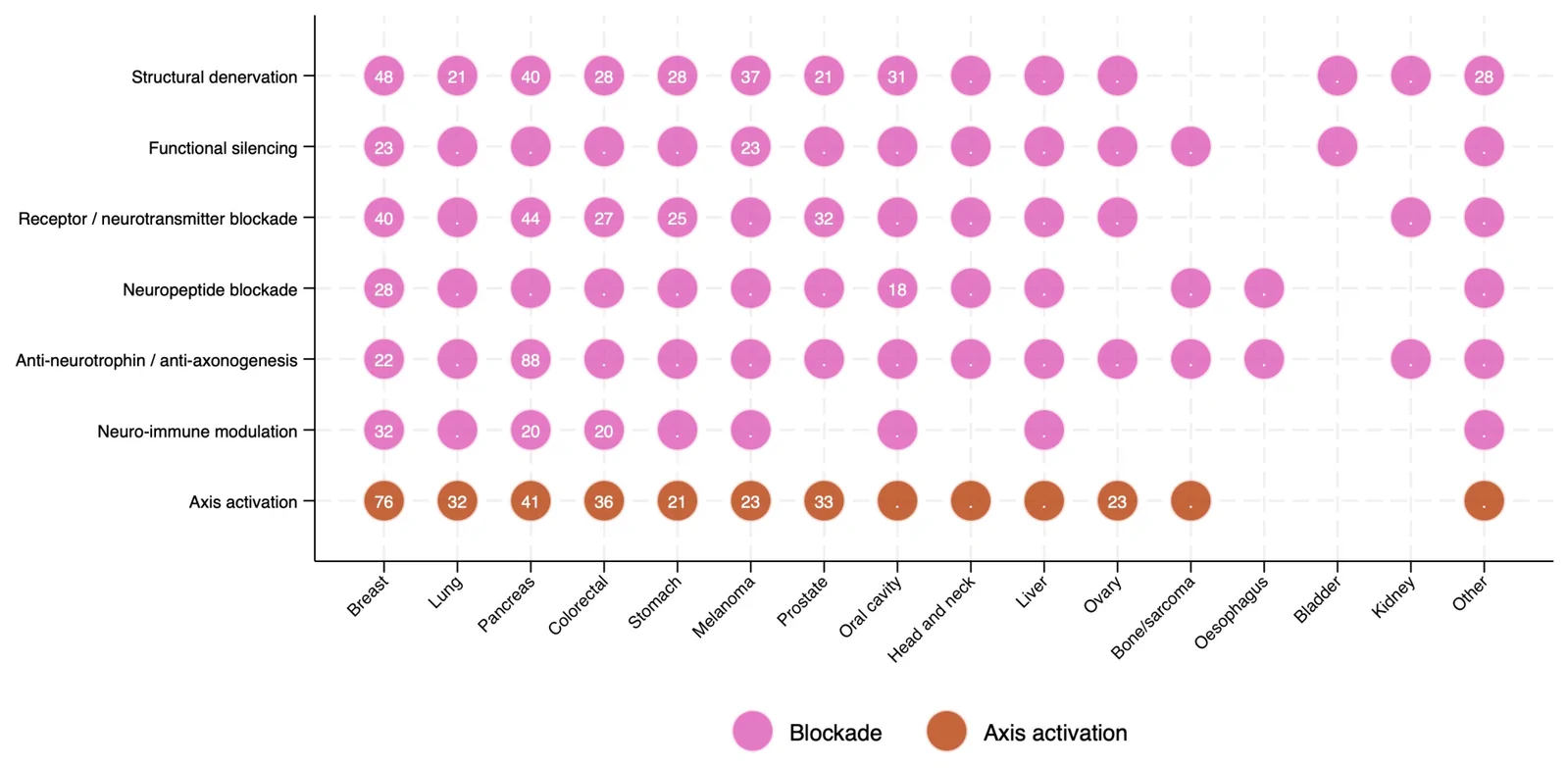
Evidence map: number of extracted effects by tumor location and level of intervention. Bubble area is proportional to the number of effects. Brown, interventions that activate the axis.

The 1437 effects are distributed across tumor types, intervention levels, and nerve fibers, as shown in the evidence map (Figure 3). The timing of intervention is strongly unbalanced: of the 1107 effects in the model, 509 come from interventions applied before tumor inoculation (T0), 250 from the immediate post-inoculation window (T1), and 268 from interventions on already established tumors (T2 or T3). In other words, the preclinical literature in this field predominantly tests prevention, not treatment.

The reported variance was the standard error of the mean in most cases, which necessitated systematic conversion to standard deviation; a significant number of effects reported no dispersion at all, and in the older literature we found the group size to be frequently missing.

The complete distribution of evidence across the three classifications—study arm, nerve fiber, molecular system, timing, immune status, route of administration, target engagement, and data source—is given in Table 1.

### 3.3. The Global Effect of Axis Blocking

Of the 1437 extracted effects, 160 had their mean, variance and power reported directly in the text, table or source data file. The remaining values were obtained by digitizing the graphic panels, with calibration on the *Y*-axis of each panel and cropping at increased resolution where the error bar was not legible at the page resolution; each digitized value was given a confidence score. This resulted in 1107 effects from 986 experiments and 297 articles entering the model—552 with high confidence, 298 with medium confidence and 97 with low confidence. The effects left out of the model are those with outcomes outside the prespecified hierarchy, those whose panels were in supplementary materials that we could not obtain, and those with numbers below the calculation threshold.

For 26 experimental arms, the figure did not contain any error bars or individual points from which to estimate dispersion; for these, the standard deviation was imputed from the median coefficient of variation of effects at the same level of the outcome hierarchy. The sensitivity analysis excluding them is reported in Section 3.10.

Blocking the peripheral nerve–tumor axis reduced tumor burden by −44.8% (95% confidence interval, 95% CI: −48.3 to −40.9), on 854 effects from 754 experiments and 266 articles (*p* < 0.001). Heterogeneity was high, I^2^ = 97%, distributed between the article level (τ^2^ = 0.161) and the experiment level (τ^2^ = 0.242), which justifies the three-level structure.

### 3.4. Effect on Molecular Signaling Systems

The six levels of intervention describe the method by which the axis is interrupted—denervation, silencing, and pharmacological blockade. However, they do not say which signaling system was engaged: a chemical sympathectomy and a β-blocker use different methods but reach the same noradrenergic pathway. We therefore classified, post hoc, each of the 1291 experiments according to the molecular system that the intervention interrupts or activates, regardless of the method. The classification is exploratory and does not appear in the protocol; we report it as such.

**Figure 4:**
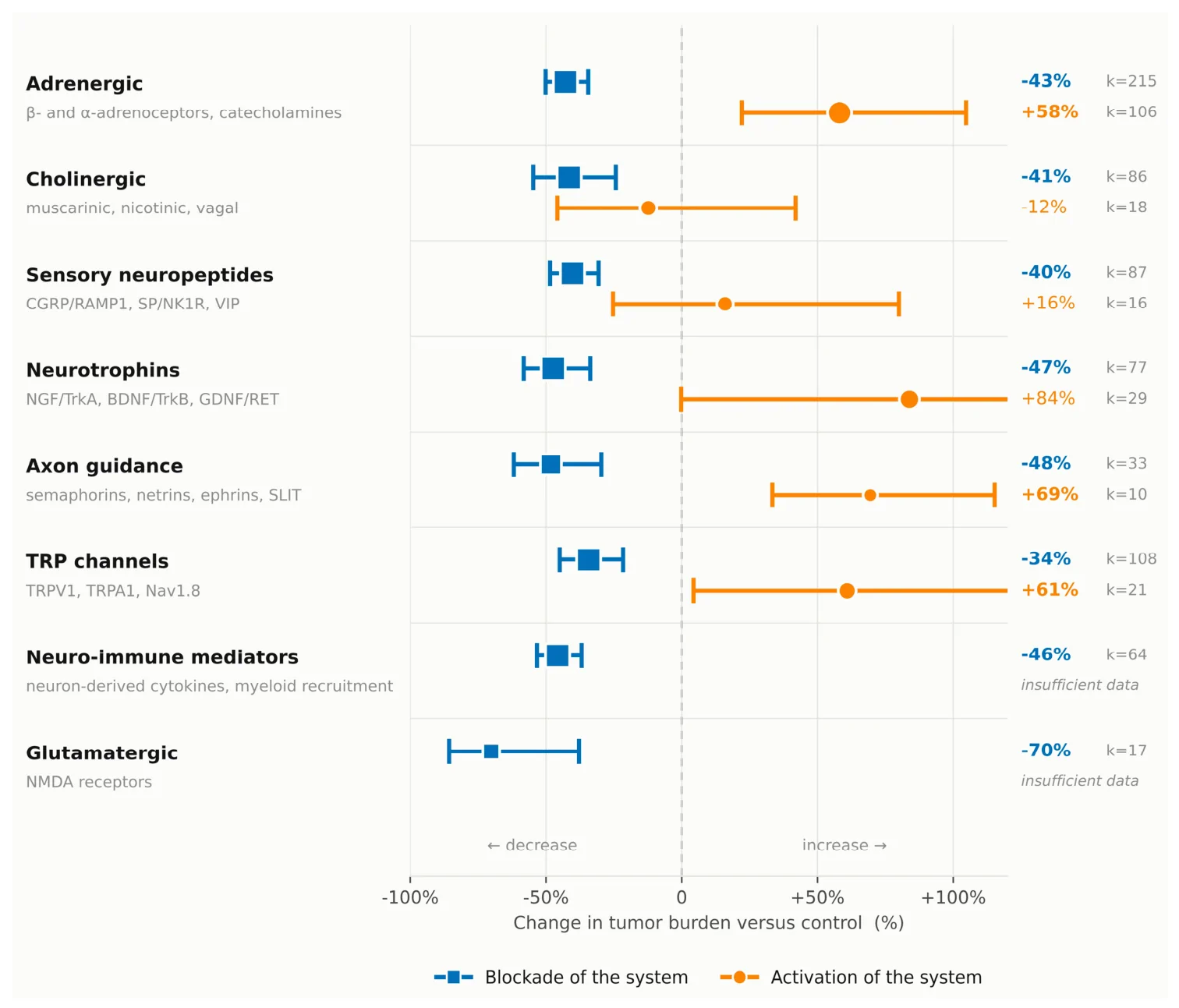
Effect by molecular signaling system, blockade versus activation. Post hoc classification of every experiment by the signaling system engaged; blockade and activation were fitted as separate three-level models, without sign inversion. Squares, blockade; circles, activation. Bars are 95% confidence intervals.

The results are shown in Figure 4 and Table 2. All eight systems respond to blockade, with estimates ranging from −34.3% (95% CI: −44.9 to −21.6) for transient receptor potential (TRP) channels to −70.2% (95% CI: −85.7 to −37.8) for glutamatergic signaling. Seven of the eight fall within a band of approximately fifteen percentage points, between −34% and −49%; the only exception is the glutamatergic compartment. Meta-regression with the adrenergic system as the reference reveals no significant differences between them except for the glutamatergic compartment (*p* = 0.014), but it was estimated on 17 effects from five articles—too few to support a claim of superiority.

#### 3.4.1. Adrenergic System

This is the most well-represented system: 215 effects from 80 articles for blockade and 106 effects from 38 articles for activation. Blockade reduces tumor burden by −42.8% (95% CI: −50.1 to −34.4), and activation increases it by 58.1% (95% CI: 22.1 to 104.7) (*p* < 0.001). It is the only pair in the entire analysis in which both directions are estimated on hundreds of effects, and the contrast constitutes the strongest internal evidence of the paper.

Sympathetic fibers act predominantly through β-adrenergic receptors. β2 signaling promotes angiogenic switching, metabolic remodeling, and the expansion of myeloid-derived suppressor cells [5,68,69,70,71], and β3 receptors contribute to the organization of the microenvironment [72,73]. Catecholamines stimulate angiogenesis and lymphangiogenesis [74,75,76,77,78] and induce depletion of cytotoxic T lymphocytes [79,80].

Chemical or surgical sympathectomy reduces tumor growth in most models [81,82,83,84,85,86], although negative or contextual results have also been reported [87,88,89]. The sympathetic component also contributes to premetastatic niche formation and bone metastasis [90,91,92]. Innervation dependence has been most clearly documented in the prostate [93,94,95,96] and integrated into a self-sustaining adrenergic–neurotrophin loop [97,98].

#### 3.4.2. Cholinergic System

Blockade of cholinergic signaling—vagotomy, muscarinic antagonists, deletion of the muscarinic M3 receptor gene (cholinergic muscarinic receptor 3, CHRM3)—reduces tumor burden by −41.4% (95% CI: −54.7 to −24.3) across 86 effects from 30 articles, a magnitude virtually identical to that of adrenergic blockade. Activation, however, does not produce the opposite effect: the estimate is −12.3% (95% CI: −45.8 to 41.9), nonsignificant (*p* = 0.593) and with a confidence interval covering both a substantial decrease and an increase. On 18 effects, the correct conclusion is not that cholinergic activation is protective, but that we have no evidence that it is a promoter—the only system in the analysis for which we can say this.

Muscarinic signaling supports gastric and intestinal tumorigenesis [6,7,8,99,100,101,102,103], and vagotomy modifies gastric tumor progression in both animal models and human cohorts [104,105,106,107,108,109,110]. Non-neuronal acetylcholine, produced by tumor or immune cells, adds an autocrine loop [111,112,113,114]. The cholinergic anti-inflammatory pathway links vagal tone to immunosurveillance [115,116,117,118,119,120].

#### 3.4.3. Sensory Neuropeptides

Blockade of neuropeptides released by sensory fibers—CGRP and its receptor activity-modifying protein 1 (RAMP1), substance P, neurokinin 1 receptor (NK1R), and VIP—resulted in a −40.2% (95% CI: −48.5 to −30.6) reduction in 87 effects from 35 articles. Activation, on 16 effects, gives 15.9% (95% CI: −25.3 to 80.0), without statistical significance (*p* = 0.510).

Sensory neurons release CGRP, substance P, VIP, galanin, neuromedin U and pituitary adenylate cyclase-activating polypeptide (PACAP) into the tumor microenvironment [9,10,11,12,121,122,123,124,125,126,127,128,129,130,131,132,133]. Pharmacological blockade of these pathways—with anti-CGRP antibodies, NK1R antagonists or capsaicin—reduces tumor growth in several models [134,135,136,137].

However, the role of nociceptors is ambivalent: the same neurons can suppress immunosurveillance or, on the contrary, support it, depending on the model and the subpopulation targeted [138,139,140,141,142,143,144,145,146,147,148]. This ambivalence explains the extreme heterogeneity observed in the sensitive subgroup of the meta-analysis.

#### 3.4.4. Neurotrophins and Trk Receptors

Blockade of neurotrophins and their receptors produces one of the largest reductions in the series, −47.4% (95% CI: −58.2 to −33.7) across 81 effects from 34 articles. Exogenous administration of neurotrophins goes in the opposite direction, with a large point estimate—83.8% (95% CI: −0.2 to 238.7)—but it is imprecise and borderline significant (*p* = 0.051, 29 effects). The direction is consistent with blockade; the magnitude cannot be stated.

Tumor axonogenesis is driven by the neurotrophins nerve growth factor (NGF), brain-derived neurotrophic factor (BDNF), glial cell line-derived neurotrophic factor (GDNF), and artemin, through the tyrosine kinase receptors TrkA and TrkB (tropomyosin receptor kinase A and B, respectively), RET (rearranged during transfection), and the neurotrophin receptor p75 (p75NTR) [149,150,151,152,153,154,155,156,157,158,159,160,161,162,163,164]. Classical axon guidance molecules—semaphorins, netrins, ephrins, and SLIT–ROBO (slit guidance ligand–roundabout receptor)—are reused by the tumor to organize its innervation [165,166,167,168,169,170,171,172,173]. Tumor extracellular vesicles carry signals that reprogram neurons at a distance [174,175,176,177,178,179,180,181,182].

Clinically approved TRK inhibitors act on the same family of receptors, but in the indication of oncogenic fusions of the NTRK (neurotrophic tyrosine receptor kinase) genes, which is why they were excluded from the present synthesis [183,184].

#### 3.4.5. Axonal Guidance Molecules

Interference with semaphorins, netrins, ephrins and the SLIT–ROBO axis reduces tumor burden by −48.2% (95% CI: −61.9 to −29.6) on 33 effects, and overexpression or administration of these molecules increases it by 69.5% (95% CI: 33.4 to 115.3) (*p* < 0.001). It is the second bidirectional pair with both arms significant, although on much smaller volumes than the adrenergic one.

#### 3.4.6. TRP Channels of the Nociceptor

Ablation or blockade of TRPV1+ (transient receptor potential vanilloid 1) and TRPA1+ (transient receptor potential ankyrin 1) neurons—by capsaicin, resiniferatoxin, or genetic models—yields −34.3% (95% CI: −44.9 to −21.6) on 108 effects, the lowest magnitude of the series. Activation of the same channels increases tumor burden by 60.9% (95% CI: 4.3 to 148.2) (*p* = 0.031). Bidirectionality is also verified here, but the amplitude of the blockade is noticeably smaller than in the other systems, which is consistent with the functional ambivalence of nociceptors described below.

#### 3.4.7. Neuro-Immune Mediators

Interventions targeting neuro-immune mediators yielded −45.7% (95% CI: −53.3 to −36.9) on 64 effects from 23 articles. There are not enough activation studies in this category for a separate estimate.

Nerve fibers shape the composition of the immune infiltrate, the formation of tertiary lymphoid structures, and the response to checkpoint blockade [185,186,187,188,189,190,191,192,193,194,195,196,197,198]. This interface explains why the effect of axis blockade is maintained in immunodeficient hosts, but with different magnitudes.

#### 3.4.8. Perineural Invasion and the Perineural Niche

Perineural invasion is not a separate signaling system, but an anatomical context in which several of the above systems act simultaneously. The effects that measure it continuously have entered the analysis at level 5 of the outcome hierarchy, through amendment 1, and are distributed between the neurotrophic, adrenergic and neuropeptide systems.

Perineural invasion is documented as a prognostic factor in pancreatic, gastric, esophageal, biliary, periampullary, oral, rectal, colorectal and renal cancers [15,16,17,199,200,201,202,203,204,205,206,207,208,209,210]. The central mechanism is the dialogue between the tumor cell and the Schwann cell, which dedifferentiates and guides tumor migration along the nerve [14,211,212,213,214,215,216,217]. Enteric glia play an analogous role in colorectal cancer [218,219,220,221,222,223].

### 3.5. Effect by Intervention Level

**Figure 5:**
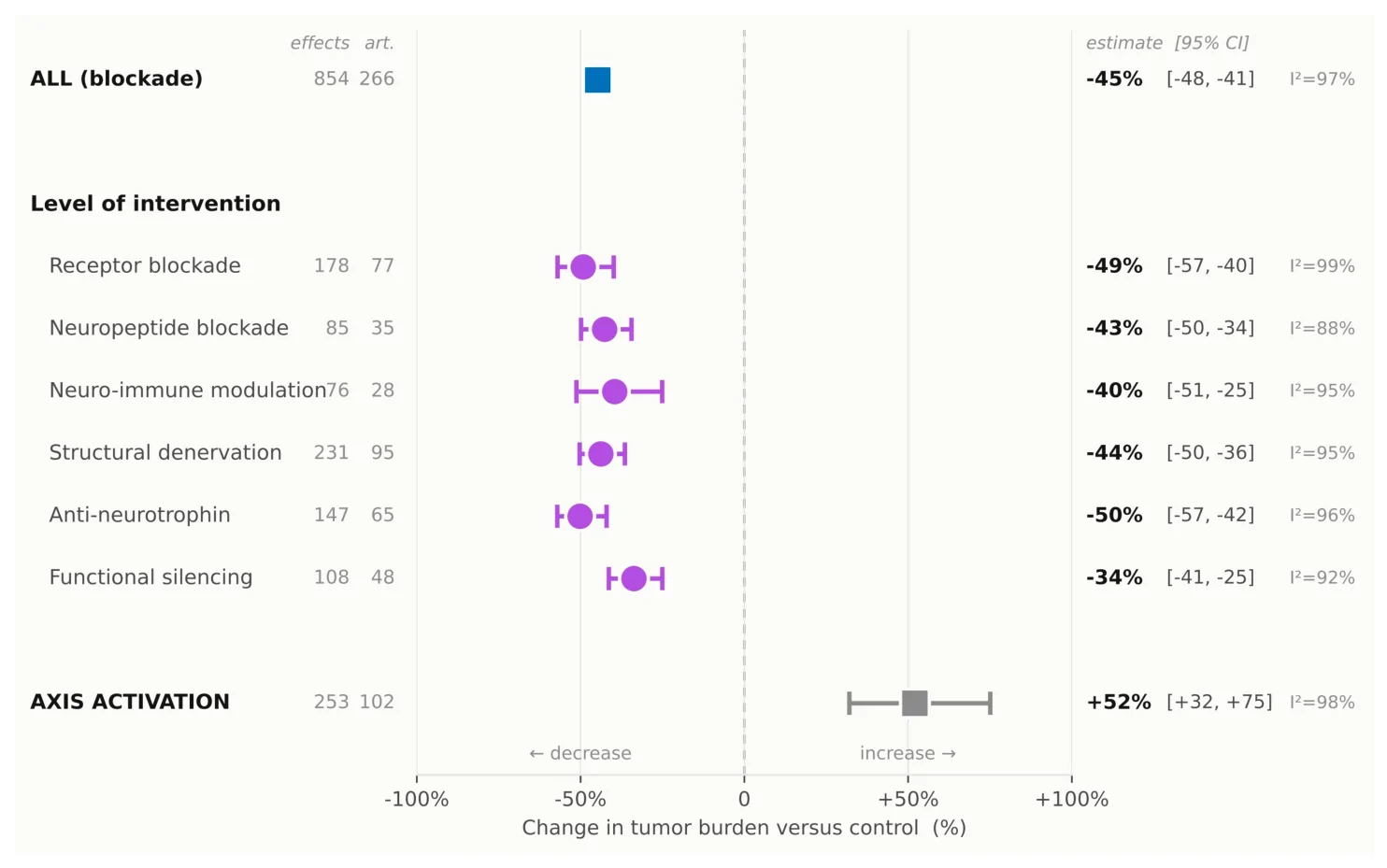
Effect of blocking the peripheral nerve–tumor axis, by level of intervention. Three-level random-effects model (effect/experiment/article), REML, log response ratio. Marker area is proportional to the number of effects.

A complementary classification, by the method by which the axis is disrupted, is shown in Figure 5 and Table 3. All six levels produce significant reductions. The largest effects are for blockade of neurotrophins and axonogenesis (−50.1% (95% CI: −57.1 to −42.1), 151 effects) and blockade of the receptor or neurotransmitter (−49.2% (95% CI: −57.0 to −39.9), 178 effects). Structural denervation, the most frequently studied approach, yields −43.8% (95% CI: −50.3 to −36.5) on 231 effects, neuropeptide blockade −42.7% (95% CI: −49.9 to −34.4), neuro-immune modulation −39.6% (95% CI: −51.3 to −25.1), and functional silencing −33.7% (95% CI: −41.4 to −25.0)—the smallest effect of the series, but significant (*p* < 0.001).

The convergence of the two classifications is itself a result: whether we group the studies by the molecular system employed or by the method of interruption, the magnitudes converge in the same band. The common target is not a molecule or a technique, but the axis as a signaling system.

### 3.6. Primary Analysis: Established Tumor Intervention

Restricting to effects at time point T2 or T3—intervention applied to an already measurable tumor, i.e., the clinically relevant window—retained 221 effects from 173 trials and 81 articles. The reduction in tumor burden was −52.0% (95% CI: −58.4 to −44.6) (*p* < 0.001), with I^2^ = 98%.

**Figure 6:**
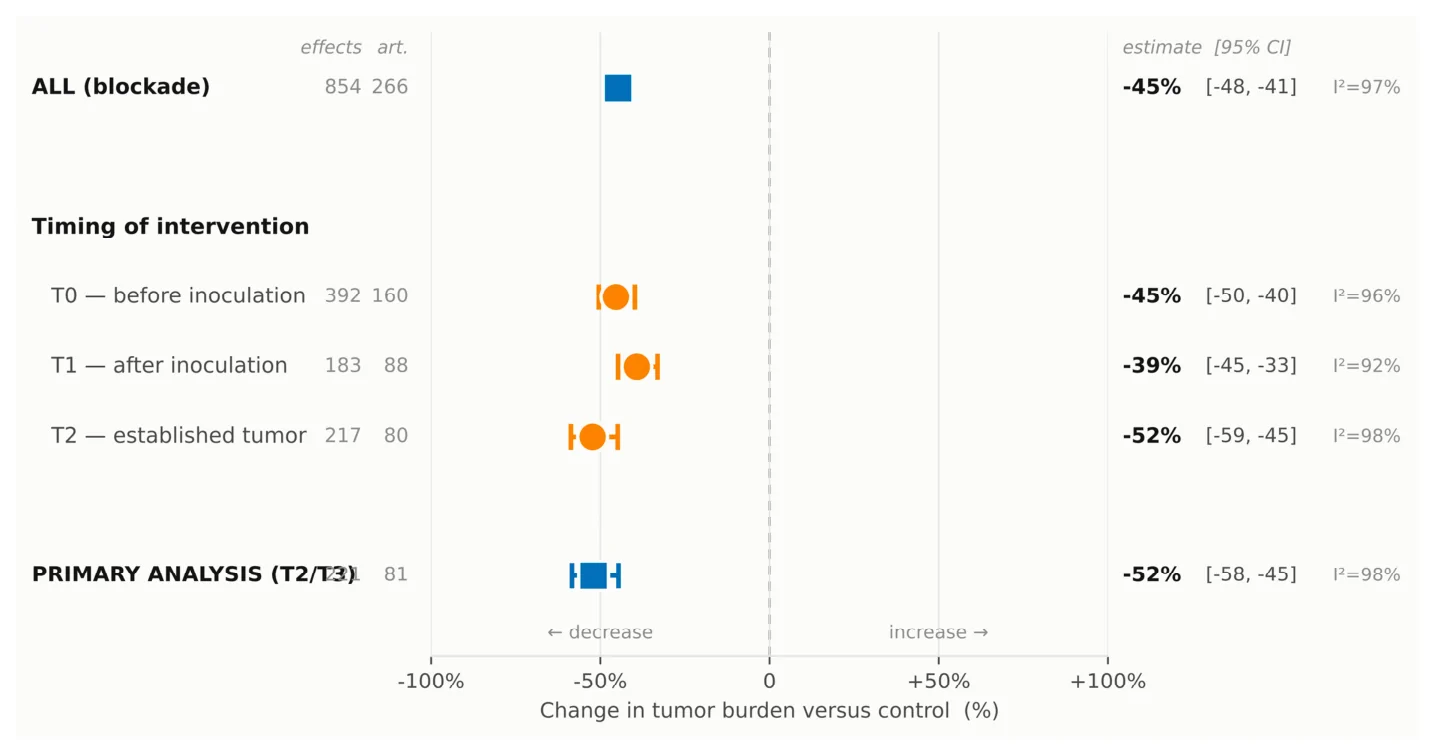
Timing of the intervention relative to tumor stage. The moderation test compares the therapeutic window (T2/T3) with the preventive window (T0/T1) within the same three-level model.

In comparison, interventions applied before tumor inoculation yielded −45.4% (95% CI: −50.5 to −39.8), and those in the immediate post-inoculation window yielded −39.2% (95% CI: −44.8 to −33.1) (Figure 6). The moderation test did not reveal a significant difference between the therapeutic and preventive windows (log response ratio, lnRR: −0.091; *p* = 0.17). Contrary to expectation, the effect in the therapeutic window is not smaller than that in the preventive window, but numerically slightly larger—a result that, although insignificant, does not support the hypothesis that axis blocking would work only prophylactically.

### 3.7. Prespecified Subgroup Analyses

**Figure 7:**
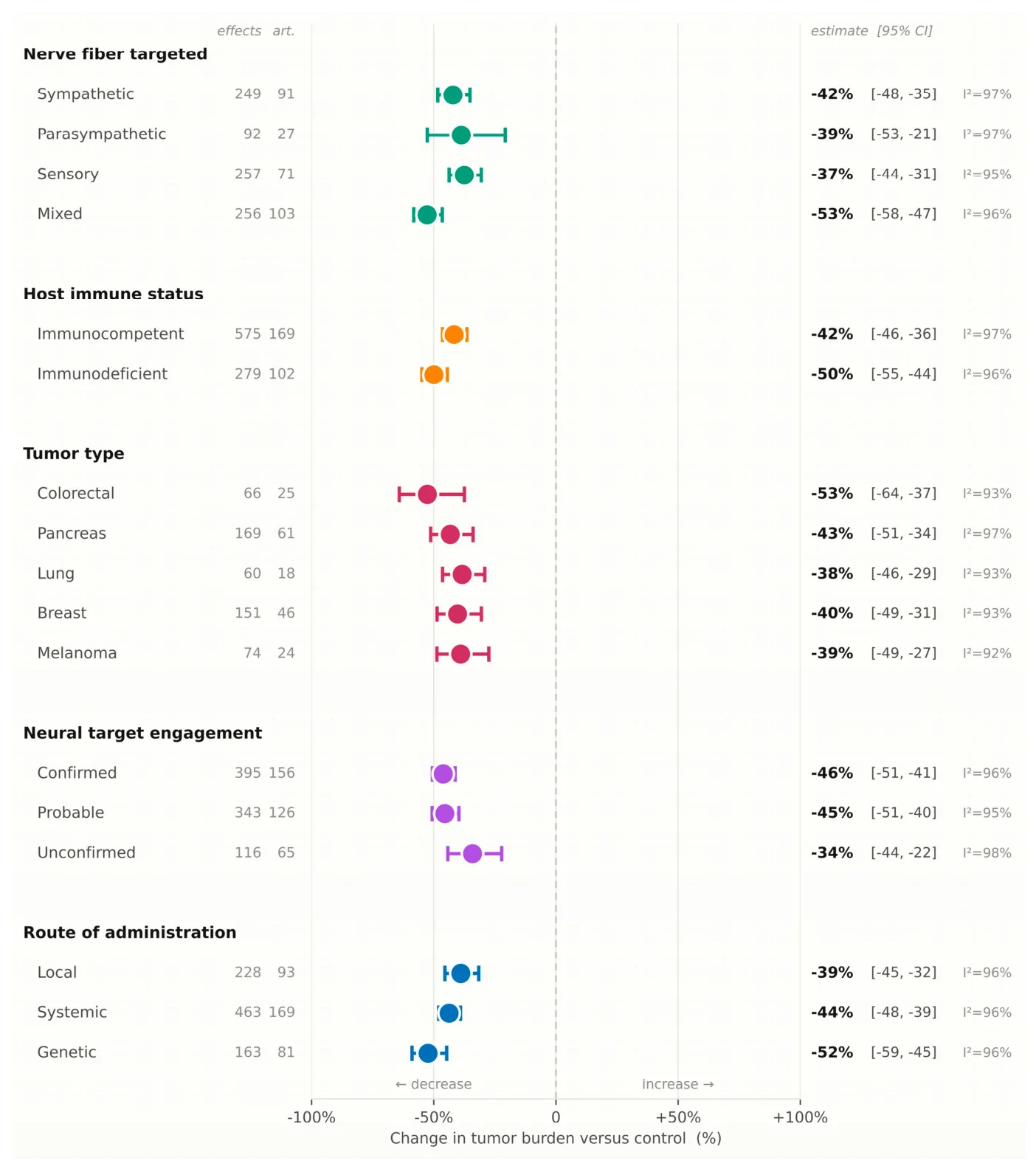
Prespecified subgroup analyses. Blockade studies only, outcomes from hierarchy levels 1–5.

The results for the prespecified subgroups are presented in Figure 7 and Table 3.

Nerve fibers. The effect was present in all four categories: sympathetic fibers: −42.1% (95% CI: −48.3 to −35.2) (249 effects, I^2^ = 97%), parasympathetic fibers: −38.8% (95% CI: −52.7 to −20.8), sensory fibers: −37.5% (95% CI: −43.7 to −30.5) (257 effects), and mixed fibers: −52.7% (95% CI: −58.2 to −46.5). Meta-regression with sympathetic fibers as a reference revealed no significant differences between compartments (minimum *p* = 0.050). The sensory compartment, which in the preliminary analysis of the subset with values reported in the text seemed to be unresponsive, turns out to be as sensitive as the others after completing the database; however, its heterogeneity remains among the highest (I^2^ = 95%).

Neural target engagement. Studies that actually documented changes in innervation, neuronal activity, or local neurotransmitter concentration reported −46.1% (95% CI: −50.6 to −41.3); those with only probable engagement reported −45.5% (95% CI: −50.7 to −39.7); and those without any target verification reported −34.1% (95% CI: −44.2 to −22.2). The effect is not greater where the target was not verified, but smaller and more imprecise, with the highest heterogeneity of the series (I^2^ = 98%)—a pattern that does not support the hypothesis that lack of verification would systematically inflate estimates.

Immune status. The effect was present in both immunocompetent hosts (−41.7% (95% CI: −46.4 to −36.4)) and immunodeficient hosts (−49.9% (95% CI: −54.9 to −44.5)), indicating an antitumor component that does not depend exclusively on adaptive immunity.

Route of administration. Local administration produced −38.9% (95% CI: −45.4 to −31.6), systemic administration −43.7% (95% CI: −48.0 to −39.1), and genetic manipulation −52.3% (95% CI: −58.9 to −44.7).

Tumor type (Table 4). The effect was present in all locations with sufficient data. The largest reductions were observed in prostate (−58.7% (95% CI: −71.6 to −39.8)), colorectal (−52.6% (95% CI: −64.1 to −37.5)) and gastric cancers (−53.4% (95% CI: −61.4 to −43.9)), followed by oral cavity (−46.7% (95% CI: −59.2 to −30.4)), pancreas (−43.3% (95% CI: −51.3 to −33.9)), breast (−40.3% (95% CI: −48.7 to −30.5)), melanoma (−39.0% (95% CI: −48.7 to −27.5)) and lung cancers (−38.4% (95% CI: −46.4 to −29.1)). The only location with a non-significant estimate was the liver (−10.9% (95% CI: −40.2 to 32.8), only 16 effects), for which the volume of data was the smallest.

Host species and sex. The included literature is almost exclusively murine: of the 351 articles that contributed extractable data, 332 (94.6%) used mice, 17 (4.8%) used rats and two (0.6%) used another species. The sex of the host was reported in 256 articles (72.9%)—male only in 105 (29.9%), female only in 97 (27.6%) and both sexes in 54 (15.4%)—and was not reported in 95 (27.1%). Strain and age were specified in the extraction form but were not retained as structured variables. Fitted as a moderator in the same three-level model, host sex was not significant overall (Q = 6.29, df = 3, *p* = 0.098), although the female-only versus male-only contrast was nominally so: −37.5% (95% CI: −44.2 to −29.9; 226 effects, 72 articles) against −49.0% (95% CI: −55.5 to −41.6; 207 effects, 78 articles). This contrast is confounded with tumor site, because the hormone-dependent models are sex-restricted by construction: 116 of the 151 breast effects come from female-only hosts and all 52 prostate effects from male-only hosts, and these two sites lie at opposite ends of the distribution (breast: −40.3%, 95% CI: −48.7 to −30.5; prostate: −58.7%, 95% CI: −71.6 to −39.8). Excluding breast, prostate and ovarian models and refitting sex on the remaining 634 effects from 205 articles, the association disappeared (Q = 2.34, df = 3, *p* = 0.505; female versus male *p* = 0.304). The 21 effects contributed by rats gave −65.5% (95% CI: −81.4 to −35.7) against −44.3% (95% CI: −47.9 to −40.3) for mice, on too few articles to interpret. The full stratification is given in Supplementary Table S11.

### 3.8. Bidirectionality of the Axis

The 253 effects from interventions that increase neural signaling, from 102 articles, produce an aggregate increase in tumor burden of 52.1% (95% CI: 32.1 to 75.1) (*p* < 0.001), with maximum heterogeneity (I^2^ = 98%). The two arms were modeled completely separately, without sign reversal and without sharing any effect.

However, analysis of molecular systems shows that bidirectionality is not uniform, and the correct formulation is more nuanced than the global contrast. This is demonstrated in three systems, in which both arms are significant and of opposite direction: adrenergic, axon guidance and TRP channels. It is suggested but unconfirmed for neurotrophins, where the activation estimate is large but imprecise and stops right at the threshold of significance. And it is absent for sensory neuropeptides and the cholinergic system, where activation produces no detectable increase.

This structure matters for interpretation. If the axis were to function as a linear switch, all systems should behave the same in both directions. They do not. Most likely, the systems differ in how close to saturation they are under basal conditions: a system that is already maximally active cannot be further stimulated, although it can be blocked. The adrenergic system, which responds robustly in both directions, appears to be the furthest from saturation—which is consistent with the fact that basal sympathetic tone in the laboratory animal is experimentally modulated by something as trivial as housing temperature.

### 3.9. Risk of Bias

**Figure 8:**
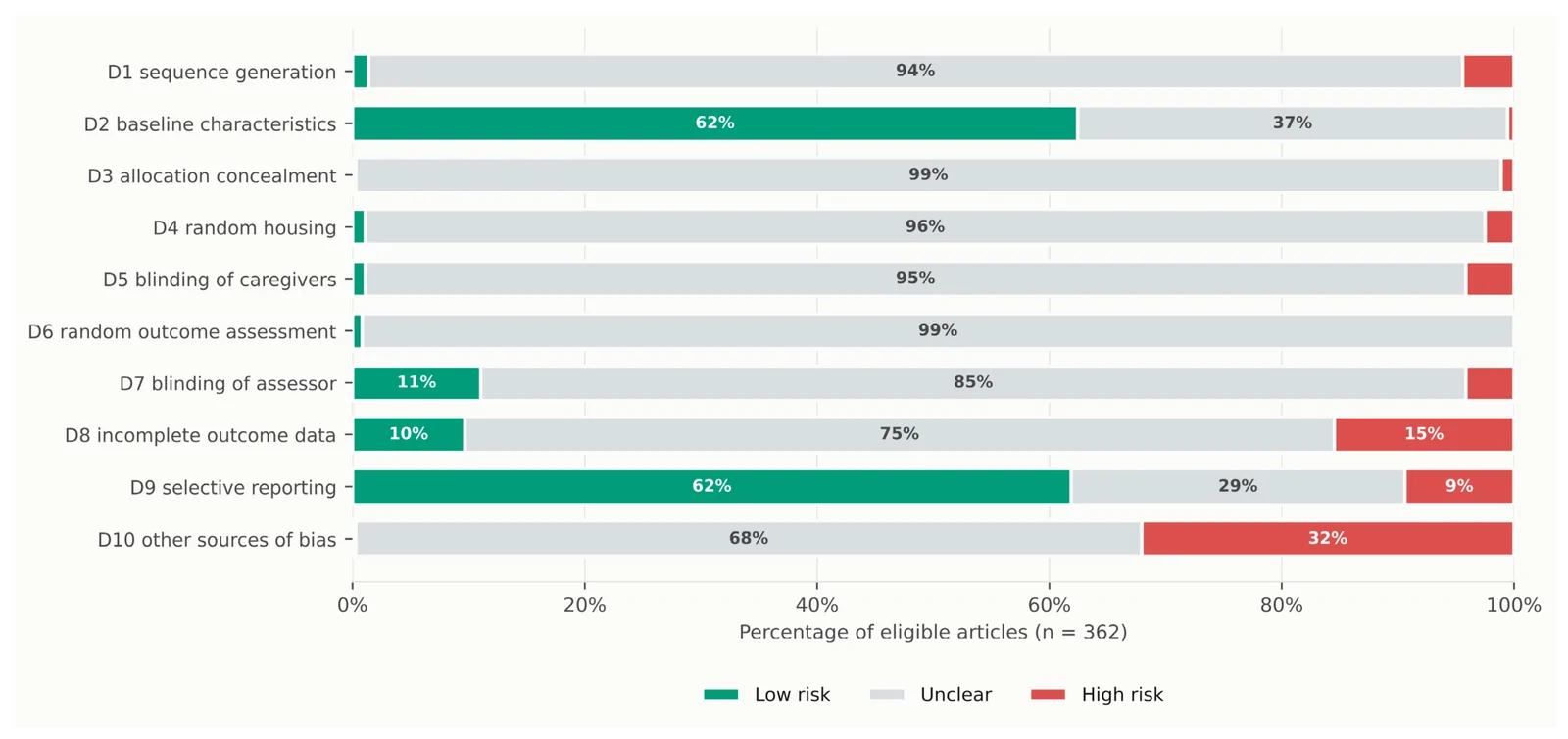
Risk of bias assessed with the SYRCLE tool across all 362 eligible articles.

The SYRCLE (Systematic Review Centre for Laboratory Animal Experimentation) assessment of the 362 eligible articles shows a pattern characteristic of the preclinical literature (Figure 8, Table 5). The areas related to randomization and allocation concealment are almost completely unclear—94% for allocation sequence, 98% for allocation concealment, and 99% for randomization at assessment—not because the procedures were incorrect, but because the articles are silent about them. Blinding of the outcome assessor is reported in 11% of the articles. Areas with explicit high risk are incomplete outcome data (15%) and the category “other sources of bias” (32%), the latter mainly reflecting pseudoreplication: reporting histological sections or microscopic fields as the experimental unit, instead of animals.

### 3.10. Small-Study Effects and Robustness

**Figure 9:**
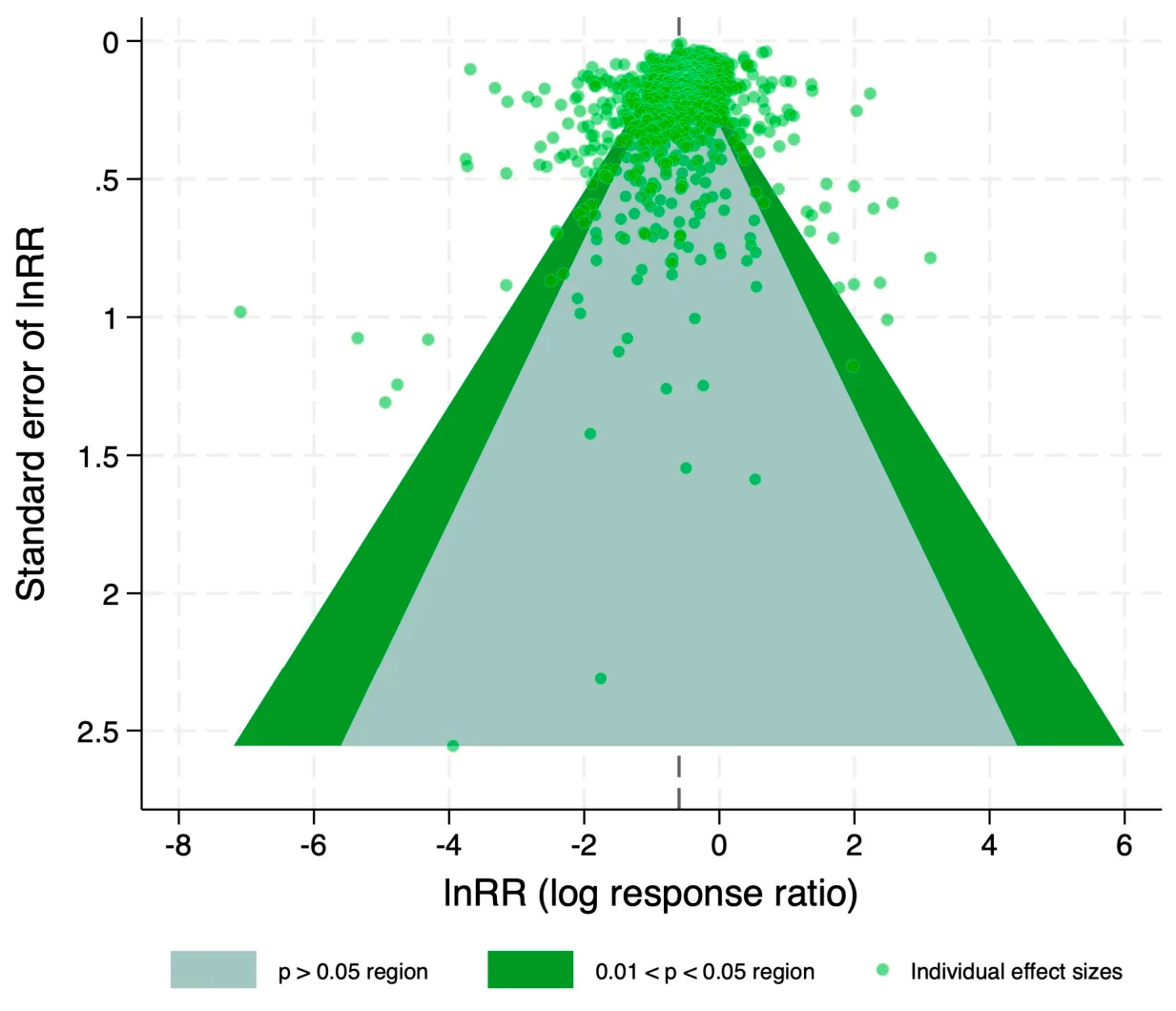
Contour-enhanced funnel plot, blockade studies. Shaded wedges mark *p* < 0.05 and *p* < 0.01.

The sensitivity analyses are summarized in Table 6. The funnel plot (Figure 9) is visibly skewed, and the Egger test adapted to the multilevel model confirms marked small-study effects: slope −1.12 (standard error, ES: 0.14), *p* < 0.001. Small studies consistently report larger effects than large ones. This pattern is common in the preclinical literature and may reflect both publication bias and true heterogeneity or differences in methodological quality; whatever the cause, caution is warranted in interpreting the aggregate effect size.

Article-level leave-one-out analysis, applied to the primary analysis, produced estimates between −52.7% and −50.7% across the 81 included articles. The estimate remained negative and significant regardless of which articles were excluded, and the overall amplitude of the variation—less than three percentage points—suggests that no single study determines the result.

We repeated the overall analysis, excluding effects with low digitizing confidence and those with imputed standard deviation. The resulting estimate was −42.6% (95% CI: −46.2 to −38.7) on 764 effects, virtually identical to the main estimate, indicating that the result does not depend on reading values under difficult conditions.

### 3.11. Chronic Stress as a Physiological Activator of the Axis

Chronic stress is the most widely used model of axis activation and accelerates digestive carcinogenesis, tumor progression, and resistance to therapy [3,224,225,226,227,228,229]. The temperature at which animals are housed, through the adrenergic tone it induces, significantly modifies experimental outcomes and resistance to immunotherapy [230,231,232].

### 3.12. Translational Perspective

The most advanced translational attempt is the repositioning of β-blockers. Preclinical studies and observational cohorts suggest benefit [233,234,235,236,237,238,239,240,241,242], and several therapeutic window studies and associations with immunotherapy have reported promising results [243,244,245,246,247,248,249,250,251,252,253]. However, the results remain discordant between analyses [254,255,256,257,258].

Celiac plexus neurolysis, the only denervation procedure currently used in practice, remains an analgesic procedure with no evidence of antitumor effect [259,260,261,262]. Heart rate variability and resting heart rate have been proposed as accessible autonomic biomarkers [263,264,265].

The current state of the art is summarized in several reference works [266,267,268,269,270,271,272,273,274,275,276,277,278,279,280,281,282,283,284,285,286], and innervation has been characterized in most tumor sites studied [287,288,289,290,291,292,293].

## 4. Discussion

This review shows that blocking the peripheral nerve–tumor axis reduces tumor burden in in vivo models of extracranial solid cancers by an aggregate magnitude of −44.8%, with an effect that is fully maintained on established tumors (−52.0%), and—the result that interests us most here—with a remarkably consistent magnitude across molecular signaling systems that have nothing in common.

A β-adrenergic antagonist, an anti-NGF antibody, an NK1R antagonist, a capsaicin that ablates TRPV1+ neurons, and interference with SLIT–ROBO signaling act on different receptors, through different transduction pathways, in different cellular compartments. They produce reductions of −42.8%, −48.5%, −40.2%, −34.3%, and −48.2%. This convergence is, in our opinion, the main result of the work.

### 4.1. Convergence: Eight Molecular Systems, One Effect

The result that we consider most important is a negative one: we did not find a privileged molecular target. Neither classification by signaling system, nor by nerve fiber, nor by intervention method produces a hierarchy. By systems, seven out of eight fall between −34.3% and −48.5%; by fibers, between −37.5% and −53.0%, with a minimum *p* = 0.050 for moderation; and by intervention levels, between −33.7% and −50.7%.

The only apparent exception is the glutamatergic compartment (−70.2%, *p* = 0.014 for moderation versus adrenergic), estimated on 17 effects from five articles. We do not build anything on this observation: at this volume, a single influential study can make the difference, and the literature on glutamatergic signaling in extracranial tumors is too recent to support a claim of superiority. We report it as a hypothesis to be tested, not as a result.

Interpreting this convergence requires caution. It does not mean that all molecules are equivalent as clinical targets: the pharmacological feasibility, therapeutic window, and toxicity differ enormously between a β-blocker in use for fifty years and an anti-semaphorin antibody that has never entered the clinic. It means, instead, that the magnitude of the antitumor effect is not the argument that decides between them. If an entire field has been searching for the optimal molecular target of tumor innervation for ten years, the aggregate data say that this search is ill-posed: there is no winner.

The most economical explanation is that the systems do not act in parallel, but in series or through a common node. Neurotrophins recruit axons; axons carry adrenergic, cholinergic, and sensory endings; and these release transmitters and neuropeptides that converge on the same pathways of proliferation, angiogenesis, and immunosuppression. Interruption at any point in the chain gives a similar result because the chain is the same. This is exactly the pattern that a signaling axis produces, not a collection of independent mechanisms.

We explicitly mention that the preliminary analysis, performed on the subset of 125 blockade effects whose values were reported directly in the text, suggested the opposite—an absent effect in the sensory compartment. Completion of the database by digitizing the graphic panels overturned this conclusion. This is a concrete example of the risk of interpreting a subset defined by the reporting method: in this field, studies on sensory fibers are on average more recent and report their results predominantly graphically, so that the subset with text values was systematically depleted in this compartment.

However, heterogeneity remains high in all systems (I^2^ between 92% and 98%). Convergence of means does not mean homogeneity: within each system, studies continue to contradict each other. This is the second important finding and refers to Section 4.7.

The most likely explanation is that intratumoral sensory neurons have opposing functions depending on the context. Sensory neuropeptides have both direct protumoral effects, through CGRP and substance P [13,294], and cytotoxic lymphocyte recruitment effects; ablation of nociceptors may therefore free the tumor from an immune constraint, not just a growth stimulus [145,147]. The same ambivalence appears in the clinical correlates: the expression of substance P and CGRP in colorectal cancer varies with stage, but not monotonically [20,21].

The practical consequence for drug development is that the choice of target should be made based on pharmacological criteria—already approved molecules, tissue penetration, therapeutic window, and chronic toxicity—and not on the promise of a superior magnitude of preclinical effect. From this perspective, the adrenergic and neuropeptide sensory systems have a considerable practical advantage, because they already have drugs approved in humans, not because they would have a greater effect.

### 4.2. Bidirectionality, Verified Within the Same Molecular System

Interventions that increase neural signaling produce the opposite effect: a 52.1% increase in tumor burden (95% CI: 32.1 to 75.1), estimated independently on 253 effects from 102 articles, with no sign reversal and without sharing any effect with the blockade arm.

Molecular classification allows a much stricter test than this global contrast. In a comparison between different arms, one can always object that the interventions, models, and laboratories differ. The objection disappears when blockade and activation target the same signaling system. For the adrenergic compartment we have 215 block effects (−42.8% (95% CI: −50.1 to −34.4)) and 106 activating effects (58.1% (95% CI: 22.1 to 104.7), *p* < 0.001). Same receptor, opposite directions, opposite effects. The pattern is repeated for axon guidance molecules (−48.2% vs. 69.5%) and for TRP channels (−34.3% vs. 60.9%).

However, bidirectionality is not universal, and this should be stated equally clearly. For neurotrophins, activation gives a large but imprecise point estimate, which stops right at the threshold of significance (83.8% (95% CI: −0.2 to 238.7), *p* = 0.051, 29 effects): the direction is consistent; the magnitude cannot be stated. For the sensory neuropeptides, activation produces no detectable increase (15.9% (95% CI: −25.3 to 80.0), *p* = 0.510). And for the cholinergic system, the activation estimate is −12.3% (95% CI: −45.8 to 41.9), with a confidence interval covering both a large decrease and a large increase—on 18 effects, this means an absence of evidence that cholinergic activation is promotive, not proof that it is protective. The distinction matters, because the literature on the cholinergic anti-inflammatory pathway and vagal stimulation invites the second interpretation, and our data do not support it.

The symmetry is not perfect even where it exists. On the logarithmic scale—the only one on which the comparison makes sense—the amplitude of the blockade (0.60) exceeds that of the activation (0.42). The presentation in percentages may give the opposite impression, because a 45% reduction and a 52% increase seem comparable when they are not: the percentage change is asymmetric by construction, and a mean ratio of 0.55 and one of 1.52 are not inverses of each other. The heterogeneity in the activation arm is, moreover, the highest in the entire analysis (I^2^ = 98%). The most likely explanation is that the systems differ in how close to saturation they are under basal conditions: a system that is already maximally active can be blocked but cannot be further stimulated. This would explain why the adrenergic compartment—whose basal tone in the laboratory animal is experimentally modulated by something as trivial as the temperature of accommodation—responds robustly in both directions, while the cholinergic system does not respond to stimulation. Part of the amplitude also comes from the fact that models of chronic stress include systemic effects—glucocorticoid immunosuppression and weight loss—that are not reducible to local neural signaling [3,295].

A methodological argument for future syntheses remains: the direction of the intervention should be treated as a design dimension of the analysis, not as an algebraic sign that allows the aggregation of the two arms. If we had applied the original protocol, which provided for the reversal of the sign, the contrast that today constitutes the strongest internal argument of this synthesis would have disappeared on average.

### 4.3. The Therapeutic Window Remains Underexplored

The distribution of intervention timings is perhaps the most important descriptive finding of this review. Of the effects entered into the model, 513 come from interventions applied before tumor inoculation and only 268 from interventions on established tumors. The preclinical literature in the field overwhelmingly tests prevention.

This is not a criticism of the individual studies—a constitutional genetic intervention or surgical denervation before inoculation are often the only feasible options to demonstrate the mechanism. However, a problem of representativeness remains: the clinically relevant window is documented by about a quarter of the available material. The actual result is, however, encouraging. The effect in the therapeutic window (−52.0%) is not smaller than that in the preventive window (−45.7%), but numerically slightly larger, and the moderation test does not detect a difference (*p* = 0.17). In other words, the data do not support the hypothesis—plausible a priori—that axis blockade would only work as long as tumor innervation has not been established.

### 4.4. Clinical Relevance and Converging Human Evidence

The preclinical results summarized here are consistent with observations from human cohorts. Tumor innervation density and markers of neuroendocrine differentiation are associated with survival in colorectal cancer [18,19], and tissue catecholamine concentrations and β2-adrenergic receptor expression in adjacent enteric plexuses are prognostic factors in gastric cancer [22,23,24]. Integrative measurement of autonomic tone through heart rate variability at diagnosis predicts survival in colorectal cancer [25], suggesting that the nervous influence has a systemic dimension beyond the local microenvironment [26,296]. Observational studies of β-blockers in cancer patients have yielded discordance [236,237,238,248], which contrasts with the consistency observed preclinically. The present meta-analysis does not identify a preclinical moderator to explain this discordance: the effect is of similar amplitude across fibers, levels of intervention, tumor locations, and host immune status. The discrepancy between preclinical robustness and clinical inconsistency must therefore be sought elsewhere—in the dose and exposure achievable in humans, in the duration of treatment, in the comorbidities that motivate the prescription of β-blockers, and in the immortal-time biases of observational studies—not in the heterogeneity of the mechanism at the preclinical level.

### 4.5. Methodological Quality of the Literature

The SYRCLE review reveals a reporting deficiency, not necessarily a conduct deficiency: over 90% of the articles do not describe the allocation sequence, allocation concealment, or randomization for the assessment. This opacity makes it impossible to distinguish between well-conducted and poorly conducted studies and limits confidence in aggregate estimates.

We also observed a chronological gradient in data reusability. Papers published between 2010 and 2017 frequently report means and variances in tables; those published after 2023 put almost everything in graphs and report numerical values in online source data files. Of the 31 articles for which we searched for such files, only four actually reported them; the rest contained figure legends, supplementary methods, or clinical tables. The paradox is that, although editorial policies on data availability have strengthened, the data necessary for a quantitative synthesis have become harder to obtain, not easier. The practical consequence for this review was that only 160 of the 1107 analyzable effects could be retrieved directly as figures; the remaining approximately 86% required panel-by-panel digitization. A third methodological aspect is worth noting: of the 451 articles assessed in full text, three were retracted. No single mechanism identified all of them—PubMed indexing missed one, and text extracted from PDFs missed another, whose retraction mention existed only as an image watermark. Verification of retractions in preclinical syntheses should at least combine bibliographic indexing, full text, and visual inspection of the page.

### 4.6. Limitations

First: reliance on graphical digitization. Only 160 of the analyzed effects came from reported values; the rest were read from graphical panels. Digitization introduces measurement error that cannot be eliminated: the axis calibration is accurate, but the position of the error bar peak is sometimes obscured by overlapping individual points, and broken or logarithmic axes increase the risk of interpolation error. We addressed this limitation by three means: a confidence score assigned to each value at the time of reading; a sensitivity analysis excluding values with low confidence and those with imputed standard deviation (−42.6% vs. −44.8% in the main analysis); and a second analysis restricted exclusively to effects with values reported in the text (−38.5% (95% CI: −46.9 to −28.8)). The three estimates agree, indicating that digitization did not introduce a systematic bias; it remains the main source of imprecision in this synthesis.

Second: effects left outside the model. Of the 1437 extracted effects, 330 were not included in the analysis—either because the outcome is outside the prespecified hierarchy, or because the required panel is in supplementary material that we could not obtain, or because the reported numbers were below the calculation threshold. This loss is not random: articles that publish many experiments in voluminous supplementary materials are over-represented among them. The direction of their effect is unknown.

Third: incomplete retrieval of full texts. Of the 838 articles searched, 387 could not be obtained. Although most of them come from the list of uncertain articles, we cannot exclude that there are eligible studies among them, and the direction of their effect is unknown.

Fourth: heterogeneity. I^2^ exceeds 85% in most analyses. The three-level model decomposes it correctly but does not explain it.

Fifth: small-study effects. The asymmetry of the funnel plot is marked and statistically confirmed. We cannot distinguish between publication bias, true heterogeneity, and differences in methodological quality.

Sixth: reporting bias. The systematic conversion from standard error to standard deviation, the inferred effects from legends, and unreported pseudoreplication in the original articles introduce an imprecision that no statistical method can correct.

### 4.7. Directions for Future Research

We observed that eight distinct molecular signaling systems produce reductions of similar magnitude. An additional preclinical study demonstrating that yet another neural receptor can be blocked with antitumor effect adds little to what is known. The useful question is no longer which molecule, but in what context and in which animal. This is an important direction for research. Moreover, we have observed that the same intervention, on the same system, produces very different effects from one model to another. Here lies the untapped information, and access to it requires systematic reporting of variables that are currently omitted: accommodation temperature, basal sympathetic tone, and innervation density of the tumor model before intervention. Also, about a quarter of the material applies interventions to established tumors. The effect is maintained there, which is encouraging, but the evidence base remains thin exactly where it matters clinically. At the reporting level, systematic publication of individual values per animal would transform quantitative syntheses in this field. The four articles in this review that published such files allowed the accurate calculation of means and standard deviations, with certain effective numbers—a quality of data that graphic digitization cannot match.

## 5. Conclusions

Blockade of the peripheral nerve–tumor axis reduces tumor burden in in vivo models of extracranial solid cancers, with an aggregate magnitude of −44.8% and an effect that remains entirely within the clinically relevant therapeutic window (−52.0%). The central finding of this synthesis is, however, that the magnitude does not depend on the molecular target: eight distinct signaling systems—adrenergic, cholinergic, sensory neuropeptides, neurotrophins, axonal guidance molecules, TRP channels, neuroimmune mediators, and glutamatergic—produce comparable reductions, and alternative classifications, by nerve fiber and by intervention method, yield the same result.

Stimulation of the same systems produces the opposite effect, demonstrated within the same molecular compartment for the adrenergic axis (−42.8% upon blockade versus 58.1% upon activation), for axonal guidance molecules and for TRP channels. Bidirectionality is not universal, however: for the cholinergic system and for sensory neuropeptides, activation produces no detectable increase.

The high heterogeneity and marked small-study effects require caution in extrapolating the magnitude: the true effect size is likely smaller than the pooled estimate. The direction of the effect is, however, robust to all sensitivity analyses performed, including the exclusion of values obtained by digitization under difficult conditions and the exclusion, article by article, of each included study. The cross-sectional consistency of the result—by nerve fiber category, by intervention class, by tumor location, and by host immune status—suggests that the common target is the nerve–tumor axis as such, and the next priority for the field is to move to models that reproduce the therapeutic window and to explain the residual heterogeneity, not to search for a better molecular target.

## Figures and Tables

**Table 1:** Characteristics of the included evidence.

Characteristic	Category	Articles	Experiments	Effects in Model
Study arm	Blockade of the axis	266	754	854
	Activation of the axis	102	232	253
Nerve fiber	Sympathetic	103	311	342
	Parasympathetic	29	96	109
	Sensory	79	284	324
	Mixed/unspecified	116	295	332
Molecular system	Adrenergic	94	286	321
	Cholinergic	33	94	104
	Sensory neuropeptides	37	91	103
	Neurotrophins	41	95	106
	Axon guidance	17	43	43
	TRP channels	45	114	129
	Neuro-immune mediators	23	64	66
	Glutamatergic	5	16	18
	Mixed/unspecified	81	183	217
Timing	T0—before inoculation	186	472	509
	T1—after inoculation	101	227	250
	T2—established tumor	98	210	263
	T3—advanced disease	2	3	5
	Not reported	35	74	80
Host immune status	Immunocompetent	185	633	715
	Immunodeficient	118	353	392
Route	Local	112	260	293
	Systemic	196	540	617
	Genetic	91	186	197
Target engagement	Confirmed	185	464	502
	Probable	144	394	455
	Unconfirmed	75	128	150
Data provenance	Reported in text or table	62	142	160
	Digitized, high confidence	189	504	552
	Digitized, medium confidence	130	276	298
	Digitized, low confidence	54	93	97

Counts refer to the 1111 effects entered in the quantitative model.

**Table 2:** Pooled effect by molecular signaling system, blockade versus activation.

Molecular System	Blockade: Change in Tumor Burden, % (95% CI)	k	Articles	I^2^, %	*p*	Activation: Change, % (95% CI)	k	Articles	*p*
Adrenergic (β/α-adrenoceptors, catecholamines)	−42.8 (−50.1 to −34.4)	215	80	98	<0.001	58.1 (22.1 to 104.7)	106	38	<0.001
Cholinergic (muscarinic, nicotinic, vagal)	−41.4 (−54.7 to −24.3)	86	30	97	<0.001	−12.3 (−45.8 to 41.9)	18	9	0.593
Sensory neuropeptides (CGRP/RAMP1, SP/NK1R, VIP)	−40.2 (−48.5 to −30.6)	87	35	92	<0.001	15.9 (−25.3 to 80.0)	16	9	0.510
Neurotrophins (NGF/TrkA, BDNF/TrkB, GDNF/RET)	−47.4 (−58.2 to −33.7)	77	33	97	<0.001	83.8 (−0.2 to 238.7)	29	13	0.051
Axon guidance (semaphorins, netrins, ephrins, SLIT–ROBO)	−48.2 (−61.9 to −29.6)	33	17	94	<0.001	69.5 (33.4 to 115.3)	10	6	<0.001
TRP channels (TRPV1, TRPA1, Nav1.8)	−34.3 (−44.9 to −21.6)	108	41	96	<0.001	60.9 (4.3 to 148.2)	21	9	0.031
Neuro-immune mediators	−45.7 (−53.3 to −36.9)	64	23	92	<0.001	—	—	—	—
Glutamatergic (NMDA receptors)	−70.2 (−85.7 to −37.8)	17	5	96	0.001	—	—	—	—
Mixed/unspecified	−50.7 (−56.8 to −43.8)	167	70	95	<0.001	85.7 (46.1 to 136.0)	50	24	<0.001

Post hoc classification. Blockade and activation were fitted as separate three-level models; no sign was inverted. Systems with fewer than 10 effects in an arm are shown as “—”. Moderation across systems, reference adrenergic: minimum *p* = 0.014.

**Table 3:** Prespecified analyses.

Analysis	Change in Tumor Burden, % (95% CI)	k	Experiments	Articles	I^2^, %	*p*
Overall effect of axis blockade	−44.8 (−48.3 to −40.9)	854	754	266	97	<0.001
Primary analysis—established tumor (T2/T3)	−52.0 (−58.4 to −44.6)	221	173	81	98	<0.001
Timing T0—before inoculation	−45.4 (−50.5 to −39.8)	392	360	160	96	<0.001
Timing T1—after inoculation	−39.2 (−44.8 to −33.1)	183	169	88	92	<0.001
Timing T2—established tumor	−52.3 (−58.7 to −44.9)	217	171	80	98	<0.001
Level—structural denervation	−43.8 (−50.3 to −36.5)	231	207	95	95	<0.001
Level—functional silencing	−33.7 (−41.4 to −25.0)	108	87	48	92	<0.001
Level—receptor/neurotransmitter blockade	−49.2 (−57.0 to −39.9)	178	153	77	99	<0.001
Level—neuropeptide blockade	−42.7 (−49.9 to −34.4)	85	76	35	88	<0.001
Level—anti-neurotrophin/anti-axonogenesis	−50.1 (−57.1 to −42.1)	147	137	65	96	<0.001
Level—neuro-immune modulation	−39.6 (−51.3 to −25.1)	76	67	28	95	<0.001
Fiber—sympathetic	−42.1 (−48.4 to −35.2)	249	220	91	97	<0.001
Fiber—parasympathetic	−38.8 (−52.7 to −20.8)	92	79	27	97	<0.001
Fiber—sensory	−37.5 (−43.7 to −30.5)	257	229	71	95	<0.001
Fiber—mixed	−52.7 (−58.2 to −46.5)	256	226	103	96	<0.001
Host—immunocompetent	−41.7 (−46.4 to −36.4)	575	504	169	97	<0.001
Host—immunodeficient	−49.9 (−54.9 to −44.5)	279	250	102	96	<0.001
Engagement—confirmed	−46.1 (−50.6 to −41.3)	395	360	156	96	<0.001
Engagement—probable	−45.5 (−50.7 to −39.7)	343	295	126	95	<0.001
Engagement—unconfirmed	−34.1 (−44.2 to −22.2)	116	99	65	98	<0.001
Route—local	−38.9 (−45.4 to −31.6)	228	203	93	96	<0.001
Route—systemic	−43.7 (−48.0 to −39.1)	463	399	169	96	<0.001
Route—genetic	−52.3 (−58.9 to −44.7)	163	152	81	96	<0.001
Activation of the axis (separate model)	52.1 (32.1 to 75.1)	253	232	102	98	<0.001

All estimates from three-level random-effects models (effects within experiments within articles), REML.

**Table 4:** Effect by tumor location.

Tumor Location	Change in Tumor Burden, % (95% CI)	k	Articles	I^2^, %	*p*
Prostate	−58.7 (−71.6 to −39.8)	52	19	96	<0.001
Colorectal	−52.6 (−64.1 to −37.5)	66	25	93	<0.001
Stomach	−53.4 (−61.4 to −43.9)	66	21	92	<0.001
Oral cavity	−46.7 (−59.2 to −30.4)	65	22	97	<0.001
Pancreas	−43.3 (−51.3 to −33.9)	169	61	97	<0.001
Breast	−40.3 (−48.7 to −30.5)	151	46	93	<0.001
Head and neck	−42.5 (−51.3 to −32.0)	44	11	86	<0.001
Melanoma	−39.0 (−48.7 to −27.5)	74	24	92	<0.001
Lung	−38.4 (−46.4 to −29.1)	60	18	93	<0.001
Ovary	−46.9 (−57.4 to −33.7)	17	5	92	<0.001
Bone/sarcoma	−35.6 (−52.7 to −12.4)	11	4	87	0.005
Liver	−10.9 (−40.2 to 32.8)	16	6	92	0.571

**Table 5:** Risk of bias (SYRCLE), assessed across all 362 eligible articles.

Domain	Low Risk, n (%)	Unclear, n (%)	High Risk, n (%)
D1 Sequence generation	5 (1.4)	341 (94.2)	16 (4.4)
D2 Baseline characteristics	226 (62.4)	134 (37.0)	2 (0.6)
D3 Allocation concealment	1 (0.3)	357 (98.6)	4 (1.1)
D4 Random housing	4 (1.1)	349 (96.4)	9 (2.5)
D5 Blinding of caregivers	4 (1.1)	343 (94.8)	15 (4.1)
D6 Random outcome assessment	3 (0.8)	359 (99.2)	0 (0.0)
D7 Blinding of outcome assessor	40 (11.0)	307 (84.8)	15 (4.1)
D8 Incomplete outcome data	35 (9.7)	271 (74.9)	56 (15.5)
D9 Selective outcome reporting	224 (61.9)	104 (28.7)	34 (9.4)
D10 Other sources of bias	1 (0.3)	245 (67.7)	116 (32.0)

“Unclear” means the article is silent on the domain, not that the procedure was flawed.

**Table 6:** Sensitivity analyses and small-study effects.

Analysis	Change in Tumor Burden, % (95% CI)	k	Articles
Main analysis (all blockade effects)	−44.8 (−48.3 to −40.9)	854	266
Excluding low-confidence digitization and imputed SD	−42.6 (−46.2 to −38.7)	764	250
Restricted to values reported in text or table	−38.5 (−46.9 to −28.8)	125	54
Leave-one-out at article level (primary analysis)	−52.7 to −50.7	—	81 articles removed in turn

Egger test adapted to the multilevel model: slope −1.12 (SE 0.14), *p* < 0.001.

## Data Availability

All extracted data, analysis codes, and files necessary to reproduce the full results are provided in the Supplementary Materials. The review protocol is deposited as a time-stamped registration with the Open Science Framework (https://doi.org/10.17605/OSF.IO/DZH4J). The database contains, for each effect, the exact source of the value—text, table, supplementary material, or figure—so that any reader can reproduce the extraction.

## Supplementary Materials

The following supporting information can be downloaded at https://www.mdpi.com/article/10.3390/ijms27209090/s1.

## Abbreviations

The following abbreviations are used in this manuscript:

ARRIVE	Animal Research: Reporting of In Vivo Experiments
BDNF	Brain-derived neurotrophic factor
CGRP	Calcitonin gene-related peptide
CHRM3	Cholinergic receptor muscarinic 3
CRLR	Calcitonin receptor-like receptor
DOI	Digital object identifier
ES	Standard error
SE M	Standard error of the mean
GDNF	Glial cell-line-derived neurotrophic factor
GRADE	Grading of Recommendations, Assessment, Development and Evaluation
95% CI	95% confidence interval
lnRR	Log response ratio
NGF	Nerve growth factor
NK1R	Neurokinin 1 receptor
NTRK	Neurotrophic tyrosine receptor kinase (gene)
OCT	Optical coherence tomography
PACAP	Pituitary adenylate cyclase-activating polypeptide
PRISMA	Preferred Reporting Items for Systematic Reviews and Meta-Analyses
PRISMA-P	PRISMA for Protocols
PRISMA-S	PRISMA for Searching
PROSPERO	International Prospective Register of Systematic Reviews
p75NTR	p75 neurotrophin receptor
RAMP1	Receptor activity-modifying protein 1
REML	Restricted maximum likelihood
RET	Rearranged during transfection
SD	Standard deviation
SLIT–ROBO	Slit guidance ligand–roundabout receptor
SYRCLE	Systematic Review Centre for Laboratory animal Experimentation
TRK/Trk	Tropomyosin receptor kinase
TRP	Transient receptor potential
TRPA1	Transient receptor potential ankyrin 1
TRPV1	Transient receptor potential vanilloid 1
VEGF-A	Vascular endothelial growth factor A
VIP	Vasoactive intestinal peptide
