# Supplementary material for: Molecular Targeting of the Peripheral Nerve–Tumor Axis in Extracranial Solid Cancers: Eight Signaling Systems, One Effect—Systematic Review and Multilevel Meta-Analysis
Source: Int J Mol Sci. 2026 Oct 14;27(20):9090. doi: 10.3390/ijms27209090 (PMC13705328; doi:10.3390/ijms27209090)
Supplement: Supplementary file 1 [file ijms-27-09090-s001.zip › S10_PRISMA_2020_and_PRISMAS_checklist.pdf]

Supplementary Material S10. PRISMA 2020 and PRISMA-S checklist

Meta-analysis of the peripheral nerve–tumor axis in extracranial solid cancers

References are to the sections of the manuscript. Where an item does not apply to a preclinical review, or could not be fulfilled, the reason is stated explicitly — we have not marked anything as fulfilled when it was not.

S10.1. PRISMA 2020 — 27 items

#	Item	Location	Note
1	Title identifies the report as a systematic review	Title	The title contains "systematic review and multilevel meta-analysis".
2	Structured abstract	Abstract	Background, Methods, Results, Conclusions. No references, per the journal's requirement.
3	Rationale for the review	1. Introduction	
4	Objectives, with the question explicitly stated	1. Introduction	Four numbered questions at the end of the Introduction.
5	Eligibility criteria	2.2	Grouped by species, tumor location, intervention, comparator, outcome.
6	Information sources	2.3 · S2	Three databases, the run date of each, checking of reference lists.
7	Full search strategy	S2	Syntax verbatim, by database and module. **One PubMed module remains to be completed before submission** — see the note in S2.
8	Selection process	2.4 · S8	Two independent passes, an intermediate category, human adjudication of disagreements.
9	Data collection process	2.5 · S3	Extraction form piloted on 20 articles.
10a	List of outcomes and their definitions	2.2 · S3.2	A five-level hierarchy, with a step-down rule.
10b	Other variables	S3.4	Controlled vocabulary for each

#	Item	Location	Note
	collected		moderator, with observed values.
11	Risk-of-bias assessment	2.6 · 3.7 · S6	SYRCLE, 10 domains, article by article.
12	Effect measure	2.7	lnRR, with variance via the delta method; expressed as 100 × (e^lnRR − 1).
13a	Study-grouping procedure	2.7 · S3.1	Three levels: effect ⊂ experiment ⊂ article.
13b	Data preparation	2.5 · S3.3	Dispersion conversions and imputation, with sensitivity analysis.
13c	Tabular and graphical presentation	Figures 2–8	
13d	Synthesis method	2.7	Three-level random-effects model, REML.
13e	Heterogeneity analyses	2.7 · 3.3	τ^2 at each level, I^2 decomposed (Cheung).
13f	Sensitivity analyses	3.8	Digitisation confidence, imputed dispersion, the subset reported in text, leave-one-out.
14	Reporting-bias assessment	2.7 · 3.8	Funnel plot with significance contours, Egger's test adapted to the multilevel model.
15	Certainty assessment	2.6	GRADE adapted for preclinical research.
16a	Selection flow	3.1 · Figure 2	Full PRISMA 2020 diagram.
16b	Studies excluded at full text, with reason	S7	All 89, individually, with the reason recorded.
17	Characteristics of included studies	3.2 · S9	The 362 articles, with the full reference and the volume of data extracted.
18	Risk of bias, per study	S6	The 10 domains for each article.
19	Results of individual studies	S4	The full dataset, 1,437 rows, with the effect size per effect.
20a	Synthesis: characteristics of contributing studies	3.2 · 3.3	

#	Item	Location	Note
20b	Synthesis: pooled result and precision	3.3 · 3.4 · Figures 4–6	
20c	Heterogeneity	3.3 · 3.5	I^2 reported for each subgroup.
20d	Sensitivity analyses	3.8	
21	Reporting bias	3.8 · Figure 8	
22	Certainty of evidence	4.6	Discussed in Limitations.
23a	General interpretation	4. Discussion	
23b	Limitations of the evidence	4.6	Seven limitations, numbered.
23c	Limitations of the review process	4.6 · S8	Incomplete retrieval of full texts; screening reliability.
23d	Implications for practice and research	4.7	
24a	Registration and protocol number	2.1	PROSPERO4animals; the number is to be added after registration.
24b	Where the protocol can be accessed	2.1 · S1	The full protocol and the amendments.
24c	Protocol amendments	2.1 · S1	Seven amendments, with the date, reason and effect of each.
25	Funding sources	Funding	
26	Conflicts of interest	Conflicts of interest	
27	Availability of data, code and materials	Data availability statement · S4 · S5	The full dataset and the Stata code.

S10.2. PRISMA-S — search reporting

#	Item	Location	Note
1	Databases and interfaces	S2.1	PubMed (NLM), Scopus (Elsevier), Web of Science Core Collection (Clarivate).
2	Study registers	—	Not used as a source for the quantitative synthesis. ClinicalTrials.gov, ICTRP and CTIS were consulted only for the translational context in the Discussion.
3	Online and grey-literature	S2.8	bioRxiv and medRxiv, for the sensitivity analysis.

#	Item	Location	Note
	searching		
4	Checking reference lists	S2.8	The lists of included articles and of the major reviews.
5	Contacting authors	—	**Not done.** Missing data were obtained through digitisation of panels, not by direct request. This is a real limitation: 330 effects remained outside the model, and some of them might have been recoverable through correspondence.
6	Other search methods	—	None.
7	Full search strategy, verbatim	S2.4–S2.6	Reproduced for each database and module. See the note in S2 regarding the PubMed module that remains to be completed.
8	Limits applied to the search	S2.1	**None.** No restriction by language, year, or publication type. Reviews were removed at screening, not at the query stage.
9	Search filters	S2.2 · 2.3	The animal-study filter, built from terms and, in PubMed, from "animals"[Mesh:noexp].
10	Preliminary searches and validation	S2.7 · S2.8	A sentinel query run before each module; the relevance filter validated on 113 known references, with no loss.
11	Controlled vocabulary	S2.2	The three-block structure of each module.
12	Adapting syntax across databases	S2.4–S2.6	Differences — MeSH, truncation, fields — are documented for each database.
13	Date restrictions	S2.1	None.
14	Peer review of the search strategy by a second person	—	**Not done formally**, through a PRESS-type tool. The strategy was checked through the sentinel query and the set of 113 known references, but this does not replace an independent review of the syntax.
15	Total number of records	S2.1 · S2.3	33,432 gross, 16,649 unique after deduplication.
16	Deduplication	S2.9	A two-step procedure — normalised DOI, then normalised title with a one-year tolerance.

S10.3. Unfulfilled items

We gather these here so the reader does not have to search for them through the tables.

Item	What is missing	Consequence
PRISMA-S 5	Authors were not contacted for missing data	330 effects remained outside the model; some might have been recoverable
PRISMA-S 14	The search strategy was not subjected to an independent PRESS-type review	risk of incomplete search sensitivity, not formally verified
PRISMA 2020 7	The syntax of one PubMed module was not saved	to be retrieved from the PubMed history before submission
PRISMA 2020 24a	The PROSPERO number	registration is under way

S10.4. Adjacent reporting standards

The review follows, in addition to PRISMA 2020 and PRISMA-S:

- **PRISMA-P** for drafting the protocol;
- **PROSPERO4animals** for registration;
- **SYRCLE** for the risk-of-bias assessment (S6);
- **ARRIVE 2.0** as a reference for assessing the reporting quality of primary studies;
- **GRADE**, in its adaptation for preclinical research, for the certainty of the evidence.
