# Supplementary material for: Molecular Targeting of the Peripheral Nerve–Tumor Axis in Extracranial Solid Cancers: Eight Signaling Systems, One Effect—Systematic Review and Multilevel Meta-Analysis
Source: Int J Mol Sci. 2026 Oct 14;27(20):9090. doi: 10.3390/ijms27209090 (PMC13705328; doi:10.3390/ijms27209090)
Supplement: Supplementary file 1 [file ijms-27-09090-s001.zip › S1_Protocol_Amendments.pdf]

Supplementary Material S1. Protocol Amendments

Meta-analysis of the peripheral nerve–tumor axis in extracranial solid cancers

---

S1.1. Timing of the amendments

The seven amendments below were adopted by team consensus on **25 August 2026**, **after data extraction had been completed and before any statistical analysis**. None was adopted after the authors had seen a pooled estimate.

We state this timing explicitly because it determines what kind of risk the amendments introduce. An amendment adopted after extraction but before analysis cannot be influenced by the result — but it can be influenced by what extraction showed about the structure of the literature. This is the case for amendments 1, 3 and 5, and below we explain for each what specifically triggered the revision.

S1.2. Synoptic table

No.	Subject	Type	Effect on the dataset
1	Primary outcome hierarchy	Broadening	+91 effects from 36 articles
2	i.v. pulmonary retention models	Confirmation	none (10 articles remain excluded)
3	Central neuron interventions	Conditional broadening	none met the condition
4	Tumors of the nerve itself	Explicit restriction	2 articles excluded
5	Axis-activation studies	Separated into its own analysis	separate arm, 253 effects
6	Multiple reports of the same study	Deduplication rule	11 pairs resolved
7	Effects with a zero mean	Calculation rule	no effect required it

---

S1.3. The amendments, in detail

Amendment 1 — broadening the primary outcome hierarchy

What changed. A fifth level was added to the hierarchy: perineural invasion extent **measured continuously** — the length of invasion along the nerve, the volume, diameter or area of the invaded nerve, or a numeric severity score.

What was not accepted. Binary incidence of perineural invasion; sciatic nerve function score; intratumoral innervation density; count of invaded nerves per field.

The line was drawn at continuous measurement of invasion **extent**, because only this is comparable in effect-size terms with the other four levels of the hierarchy.

What triggered the revision. 60 of the 89 full-text exclusions were due to the absence of an outcome from the initial hierarchy. Among them, an entire subdomain — perineural invasion — was being eliminated systematically, even though it is methodologically coherent and central to the research question.

Effect. 91 effects recovered from 36 articles. Most came from articles **already** eligible, whose perineural invasion experiments had not been recorded; a single article became eligible in its entirety.

Risk introduced. The amendment broadens the criteria after the team had seen what the literature contained. The risk is mitigated by the fact that level 5 is the lowest in the hierarchy — a level-5 effect enters the analysis only when the article reports none of levels 1–4 — and by the fact that re-evaluation of the 69 candidate articles was done without access to any pooled estimate.

Amendment 2 — pulmonary retention models after intravenous injection

What was decided. They remain excluded (10 articles).

Reason. In these models — typically MADB106 in the rat — tumor cells are injected intravenously and pulmonary retention is quantified after a few hours. There is no primary tumor and no tumor innervation. The outcome measures NK-cell-mediated immune surveillance, i.e. a different research question from the one addressed by this review.

Note. This amendment is a confirmation, not a change. We recorded it because the decision had been contested within the team and because these models represent a substantial and long-standing literature on surgical stress and β-blockers, which would have appreciably increased the volume of data.

Amendment 3 — central neuron interventions

What changed. These are included, but **only** if the peripheral sympathetic link is demonstrated experimentally: measured intratumoral noradrenaline, quantified nerve density, or abolition of the effect by a peripheral intervention. Articles included on this basis receive the moderator “central origin”.

Effect. The five articles previously excluded on this criterion were fully re-evaluated. **None** meets the condition. The amendment did not change the dataset.

Collateral observation. Re-evaluation showed that two of the five articles are the same study: a bioRxiv preprint and the published version in *Neuron*. This finding led to amendment 6.

Amendment 4 — tumors of the nerve itself

What was decided. Schwannoma, neurofibroma and malignant peripheral nerve sheath tumors are excluded as a matter of principle (2 articles).

Reason. When the tumor **is** the nerve, the nerve–tumor relationship cannot be defined: there is no distinct neural compartment that can be manipulated independently of the tumor compartment. The criterion was implicit in the initial protocol but not explicitly stated, which produced inconsistent coding in the first extraction batches.

Amendment 5 — axis-activation studies

What changed. Interventions that **increase** the neural signal — chronic stress, exogenous catecholamines, adrenergic agonists, excitatory DREADD, exogenously administered neurotrophins — are analyzed entirely separately, **with no sign reversal**. The initial protocol called for their inclusion in the main analysis with the sign reversed.

Reason, as formulated at the time of the amendment. The direction of the effect did not appear uniform within the category: exogenously administered nerve growth factor reduces tumor growth in several studies, whereas chronic stress accelerates it. A sign reversal applied to the whole category would have produced an estimate with no biological meaning.

What the final analysis showed. The activation arm, analyzed separately, gives an increase in tumor burden of 52.1% (95% CI: 32.1 to 75.1) across 253 effects from 102 articles. The contrast with the blockade arm (−44.8%) is today the strongest internal argument of the synthesis — and would have disappeared entirely on average had the initial protocol been applied. The heterogeneity in the activation arm remains the largest in the entire analysis (I^2 = 98%), which confirms the observation that motivated the amendment: this label groups together interventions with very different mechanisms.

Amendment 6 — multiple reports of the same study

What was decided. When the same study appears in multiple reports, the full publication is retained and the conference abstract and preliminary version are excluded. The rule applies at the **study** level, not the publication level, and identification is based on the combination of tumor model × intervention × sample sizes, not on the title.

Effect. Eleven pairs identified and resolved.

Amendment 7 — effects with a zero mean in one arm

What was decided. For effects with a zero mean in one arm, where lnRR is not defined, half of the smallest non-zero value observed in that experiment is added to both arms, with a sensitivity analysis excluding the corrected effects.

Effect on the final dataset: none. After complete digitisation of the graphical panels, all control and intervention means turned out to be strictly positive, so the correction was not applied to any effect. The rule remains recorded because it was prespecified and because it would have become operative had digitisation produced zero values.

S1.4. What was NOT amended

For completeness of reporting, we record the protocol elements that were discussed and that were **kept unchanged**:

- the effect size (lnRR) and the three-level model structure;
- the prespecified primary analysis (time points T2 and T3);
- the list of moderators and the order in which they are tested;
- the significance threshold and estimation method (REML);
- the eligibility criteria regarding species, tumor location and type of intervention.
