# Supplementary material for: Molecular Targeting of the Peripheral Nerve–Tumor Axis in Extracranial Solid Cancers: Eight Signaling Systems, One Effect—Systematic Review and Multilevel Meta-Analysis
Source: Int J Mol Sci. 2026 Oct 14;27(20):9090. doi: 10.3390/ijms27209090 (PMC13705328; doi:10.3390/ijms27209090)
Supplement: Supplementary file 1 [file ijms-27-09090-s001.zip › S2_Full_search_strategies.pdf]

Supplementary Material S2. Full search strategies

Meta-analysis of the peripheral nerve–tumour axis in extracranial solid cancers

Reported in accordance with PRISMA-S. The syntax below is the one actually run, reproduced verbatim.

> **One module remains to be completed.** The syntax of module 4 for PubMed was not saved in the working files, although the resulting export exists and entered deduplication. It must be retrieved from the PubMed search history and pasted here before manuscript submission. We have not reconstructed it by approximation, because PRISMA-S requires the syntax exactly as it was run.

S2.1. Search summary

Database	Interface	Run date	Modules	Records returned
PubMed	MEDLINE via the PubMed interface (NLM)	22 August 2026	4	7,128
Scopus	Scopus (Elsevier)	23 August 2026	4	16,579
Web of Science	Web of Science Core Collection (Clarivate)	23 August 2026	4	9,725
**Gross total**				**33,432**

After deduplication on normalised DOI, then on normalised title with a one-year tolerance for the publication year, **16,649 unique records** remained: 8,466 from Scopus, 5,528 from PubMed and 2,655 from Web of Science.

No language restriction. No year restriction. No publication-type filters applied at query time — reviews and review-type articles were removed at screening, not at search.

S2.2. Modular structure

The search was built from four complementary modules, not a single query. The first three cover the three intervention families set out in the protocol; the fourth is a safety net, built on domain terminology, added to recover studies that use “cancer neuroscience” vocabulary without naming a specific intervention.

Each module has the same three-block architecture, joined by AND:

Block	Content

Block	Content
1	oncology terms (cancer, tumor, neoplasm, carcinoma, melanoma, sarcoma)
2	module-specific terms
3	animal-study filter

S2.3. Number of records per module

Module	PubMed	Scopus	Web of Science	Total
Module 1	2,113	4,647	2,716	9,476
Module 2	1,255	3,205	1,943	6,403
Module 3	420	1,750	748	2,918
Module 4	3,340	6,977	4,318	14,635
**Total**	**7,128**	**16,579**	**9,725**	**33,432**

The figures above are records returned, before any deduplication. The modules overlap deliberately, and the overlap is removed at deduplication.

S2.4. PubMed

Interface: MEDLINE via the PubMed interface (NLM), advanced search

Run date: 22 August 2026

Module 1 — Denervation and physical neuromodulation

Records returned: 2,113 · Export file: `PubMed_Modul1_2026-08-22.nbib`

```
(cancer*[tiab] OR tumor*[tiab] OR tumour*[tiab] OR neoplasm*[tiab] OR
carcinoma*[tiab] OR adenocarcinoma*[tiab] OR melanoma*[tiab] OR
sarcoma*[tiab]) AND (denervat*[tiab] OR sympathectom*[tiab] OR
vagotom*[tiab] OR neurectom*[tiab] OR axotom*[tiab] OR "nerve
crush"[tiab] OR neurolysis[tiab] OR optogenetic*[tiab] OR
chemogenetic*[tiab] OR DREADD*[tiab] OR "6-hydroxydopamine"[tiab] OR
guanethidine[tiab] OR botulinum[tiab] OR capsaicin[tiab] OR
resiniferatoxin[tiab]) AND ("animals"[Mesh:noexp] OR mice[tiab] OR
mouse[tiab] OR murine[tiab] OR rat[tiab] OR rats[tiab] OR
xenograft*[tiab] OR "in vivo"[tiab])
```

Module 2 — Neurotransmitters and neuropeptides

Records returned: 1,255 · Export file: `PubMed_Modul2_2026-08-22.nbib`

```
(cancer*[tiab] OR tumor*[tiab] OR tumour*[tiab] OR neoplasm*[tiab] OR
carcinoma*[tiab] OR adenocarcinoma*[tiab] OR melanoma*[tiab] OR
sarcoma*[tiab]) AND (innervat*[tiab] OR denervat*[tiab] OR "nerve
density"[tiab] OR "nerve fibers"[tiab] OR "nerve fibres"[tiab] OR
"tumor-associated nerves"[tiab] OR "tumour-associated nerves"[tiab] OR
```

```
"cancer neuroscience"[tiab] OR axonogenesis[tiab] OR perineural[tiab]
OR sympathetic[tiab] OR parasympathetic[tiab] OR "sensory nerves"[tiab]
OR "sensory neurons"[tiab] OR "vagus nerve"[tiab] OR vagal[tiab] OR
autonomic[tiab] OR nociceptor*[tiab] OR "neural invasion"[tiab]) AND
(adrenergic[tiab] OR catecholamine*[tiab] OR norepinephrine[tiab] OR
noradrenaline[tiab] OR propranolol[tiab] OR ADRB2[tiab] OR
cholinergic[tiab] OR acetylcholine[tiab] OR muscarinic[tiab] OR
nicotinic[tiab] OR CGRP[tiab] OR "calcitonin gene-related
peptide"[tiab] OR RAMP1[tiab] OR "substance P"[tiab] OR NK1R[tiab] OR
TRPV1[tiab] OR "vasoactive intestinal peptide"[tiab] OR galanin*[tiab]
OR "neuropeptide Y"[tiab]) AND ("animals"[Mesh:noexp] OR mice[tiab] OR
mouse[tiab] OR murine[tiab] OR rat[tiab] OR rats[tiab] OR
xenograft*[tiab] OR "in vivo"[tiab])
```

Module 3 — Neurotrophins and axonogenesis

Records returned: 420 · Export file: `PubMed_Modul3_2026-08-22.nbib`

```
(cancer*[tiab] OR tumor*[tiab] OR tumour*[tiab] OR neoplasm*[tiab] OR
carcinoma*[tiab] OR adenocarcinoma*[tiab] OR melanoma*[tiab] OR
sarcoma*[tiab]) AND (innervat*[tiab] OR denervat*[tiab] OR "nerve
density"[tiab] OR "nerve fibers"[tiab] OR "nerve fibres"[tiab] OR
"tumor-associated nerves"[tiab] OR "tumour-associated nerves"[tiab] OR
"cancer neuroscience"[tiab] OR axonogenesis[tiab] OR perineural[tiab]
OR sympathetic[tiab] OR parasympathetic[tiab] OR "sensory nerves"[tiab]
OR "sensory neurons"[tiab] OR "vagus nerve"[tiab] OR vagal[tiab] OR
autonomic[tiab] OR nociceptor*[tiab] OR "neural invasion"[tiab]) AND
(NGF[tiab] OR "nerve growth factor"[tiab] OR BDNF[tiab] OR GDNF[tiab]
OR artemin[tiab] OR neurturin[tiab] OR TrkA[tiab] OR TrkB[tiab] OR
NTRK*[tiab] OR p75NTR[tiab] OR semaphorin*[tiab] OR neuropilin*[tiab]
OR netrin*[tiab] OR ephrin*[tiab] OR SLIT2[tiab] OR ROBO1[tiab]) AND
("animals"[Mesh:noexp] OR mice[tiab] OR mouse[tiab] OR murine[tiab] OR
rat[tiab] OR rats[tiab] OR xenograft*[tiab] OR "in vivo"[tiab])
```

Module 4 — Safety net

Records returned: 3,340 · Export file: `PubMed_Modul4_2026-08-22.nbib`

> **TO BE COMPLETED.** The syntax of this module was not saved in the working files, although the resulting export (`PubMed_Modul4_2026-08-22.nbib`, 3,340 records) exists and entered deduplication. The query must be retrieved from PubMed → *Advanced* → *History and Search Details*, or from the searches saved in My NCBI, and pasted here verbatim before manuscript submission. **It has not been reconstructed by approximation**, because PRISMA-S requires the syntax exactly as it was run.

Sentinel query

Short query run before each module, to verify that the syntax had been transferred correctly into the interface. It must return the article Zhao CM et al., *Denervation suppresses gastric tumorigenesis*, Sci Transl Med 2014 (PMID 25143365).

```
denervation[tiab] AND gastric[tiab] AND tumorigenesis[tiab]
```

S2.5. Scopus

Interface: Scopus (Elsevier), Advanced document search

Run date: 23 August 2026

Module 1 — Denervation and physical neuromodulation

Records returned: 4,647 · Export file: `Scopus_Modul1_2026-08-23.ris`

```
TITLE-ABS-KEY(cancer* OR tumor* OR tumour* OR neoplas* OR carcinoma* OR adenocarcinoma* OR melanoma* OR sarcoma*) AND TITLE-ABS-KEY(denervat* OR sympathectom* OR vagotom* OR neurectom* OR axotom* OR "nerve crush" OR neurolysis OR optogenetic* OR chemogenetic* OR DREADD* OR "6-hydroxydopamine" OR guanethidine OR botulinum OR capsaicin OR resiniferatoxin) AND TITLE-ABS-KEY(mice OR mouse OR murine OR rat OR rats OR rodent* OR xenograft* OR "in vivo" OR "animal model" OR "animal models")
```

Module 2 — Neurotransmitters and neuropeptides

Records returned: 3,205 · Export file: `Scopus_Modul2_2026-08-23.ris`

```
TITLE-ABS-KEY(cancer* OR tumor* OR tumour* OR neoplas* OR carcinoma* OR adenocarcinoma* OR melanoma* OR sarcoma*) AND TITLE-ABS-KEY(innervat* OR denervat* OR "nerve density" OR "nerve fiber" OR "nerve fibers" OR "nerve fibre" OR "nerve fibres" OR "tumor-associated nerves" OR "tumour-associated nerves" OR "cancer neuroscience" OR axonogenesis OR perineural OR sympathetic OR parasympathetic OR "sensory nerve" OR "sensory nerves" OR "sensory neuron" OR "sensory neurons" OR "vagus nerve" OR vagal OR autonomic OR nociceptor* OR "neural invasion") AND TITLE-ABS-KEY(adrenergic OR catecholamine* OR norepinephrine OR noradrenaline OR propranolol OR ADRB2 OR cholinergic OR acetylcholine OR muscarinic OR nicotinic OR CGRP OR "calcitonin gene-related peptide" OR RAMP1 OR "substance P" OR NK1R OR TRPV1 OR "vasoactive intestinal peptide" OR galanin* OR "neuropeptide Y") AND TITLE-ABS-KEY(mice OR mouse OR murine OR rat OR rats OR rodent* OR xenograft* OR "in vivo" OR "animal model" OR "animal models")
```

Module 3 — Neurotrophins and axonogenesis

Records returned: 1,750 · Export file: `Scopus_Modul3_2026-08-23.ris`

```
TITLE-ABS-KEY(cancer* OR tumor* OR tumour* OR neoplas* OR carcinoma* OR
adenocarcinoma* OR melanoma* OR sarcoma*) AND TITLE-ABS-KEY(innervat*
OR denervat* OR "nerve density" OR "nerve fiber" OR "nerve fibers" OR
"nerve fibre" OR "nerve fibres" OR "tumor-associated nerves" OR
"tumour-associated nerves" OR "cancer neuroscience" OR axonogenesis OR
perineural OR sympathetic OR parasympathetic OR "sensory nerve" OR
"sensory nerves" OR "sensory neuron" OR "sensory neurons" OR "vagus
nerve" OR vagal OR autonomic OR nociceptor* OR "neural invasion") AND
TITLE-ABS-KEY(NGF OR "nerve growth factor" OR BDNF OR GDNF OR artemin
OR neurturin OR TrkA OR TrkB OR NTRK* OR p75NTR OR semaphorin* OR
neuropilin* OR netrin* OR ephrin* OR SLIT2 OR ROBO1) AND TITLE-ABS-
KEY(mice OR mouse OR murine OR rat OR rats OR rodent* OR xenograft* OR
"in vivo" OR "animal model" OR "animal models")
```

Module 4 — Safety net

Records returned: 6,977 · Export file: `Scopus_Modul4_2026-08-23.ris`

```
TITLE-ABS-KEY(cancer* OR tumor* OR tumour* OR neoplas* OR carcinoma* OR
adenocarcinoma* OR melanoma* OR sarcoma*) AND TITLE-ABS-KEY("tumor
innervation" OR "tumour innervation" OR "tumoral innervation" OR
"tumor-associated nerves" OR "tumour-associated nerves" OR
"intratumoral nerves" OR "intratumoural nerves" OR "nerve density" OR
"cancer neuroscience" OR "neuro-neoplastic" OR "nerve-tumor" OR "nerve-
tumour" OR "neuron-cancer" OR "cancer-nerve" OR "neuron-tumor" OR
axonogenesis OR neoneurogenesis OR "neo-neurogenesis" OR "perineural
niche" OR "neural invasion" OR "perineural invasion" OR innervat* OR
denervat* OR "sensory neuron" OR "sensory neurons" OR nociceptor* OR
"sympathetic nerves" OR "sympathetic innervation" OR "parasympathetic
innervation" OR "vagal innervation" OR "neural activity" OR
glutamatergic OR neuroimmune OR "neuro-immune") AND TITLE-ABS-KEY(mice
OR mouse OR murine OR rat OR rats OR rodent* OR xenograft* OR "in vivo"
OR "animal model" OR "animal models")
```

Sentinel query

Short query run before each module, to verify that the syntax had been transferred correctly into the interface. It must return the article Zhao CM et al., *Denervation suppresses gastric tumorigenesis*, Sci Transl Med 2014 (PMID 25143365).

```
"Denervation suppresses gastric tumorigenesis", Sci Transl Med 2014.
TITLE-ABS-KEY(denervation AND gastric AND tumorigenesis)
```

S2.6. Web of Science

Interface: Web of Science Core Collection (Clarivate), Advanced Search, field TS=

Run date: 23 August 2026

Module 1 — Denervation and physical neuromodulation

Records returned: 2,716 · Export file: `WoS_Modul1_partea1…partea3_2026-08-23.ris`

```
TS=(cancer* OR tumor* OR tumour* OR neoplas* OR carcinoma* OR
adenocarcinoma* OR melanoma* OR sarcoma*) AND TS=(denervat* OR
sympathectom* OR vagotom* OR neurectom* OR axotom* OR "nerve crush" OR
neurolysis OR optogenetic* OR chemogenetic* OR DREADD* OR "6-
hydroxydopamine" OR guanethidine OR botulinum OR capsaicin OR
resiniferatoxin) AND TS=(mice OR mouse OR murine OR rat OR rats OR
rodent* OR xenograft* OR "in vivo" OR "animal model*")
```

Module 2 — Neurotransmitters and neuropeptides

Records returned: 1,943 · Export file: `WoS_Modul2_partea1…partea2_2026-08-23.ris`

```
TS=(cancer* OR tumor* OR tumour* OR neoplas* OR carcinoma* OR
adenocarcinoma* OR melanoma* OR sarcoma*) AND TS=(innervat* OR
denervat* OR "nerve density" OR "nerve fiber*" OR "nerve fibre*" OR
"tumor-associated nerve*" OR "tumour-associated nerve*" OR "cancer
neuroscience" OR axonogenesis OR perineural OR sympathetic OR
parasympathetic OR "sensory nerve*" OR "sensory neuron*" OR "vagus
nerve" OR vagal OR autonomic OR nociceptor* OR "neural invasion") AND
TS=(adrenergic OR catecholamine* OR norepinephrine OR noradrenaline OR
propranolol OR ADRB2 OR cholinergic OR acetylcholine OR muscarinic OR
nicotinic OR CGRP OR "calcitonin gene-related peptide" OR RAMP1 OR
"substance P" OR NK1R OR TRPV1 OR "vasoactive intestinal peptide" OR
galanin* OR "neuropeptide Y") AND TS=(mice OR mouse OR murine OR rat OR
rats OR rodent* OR xenograft* OR "in vivo" OR "animal model*")
```

Module 3 — Neurotrophins and axonogenesis

Records returned: 748 · Export file: `WoS_Modul3_2026-08-23.ris`

```
TS=(cancer* OR tumor* OR tumour* OR neoplas* OR carcinoma* OR
adenocarcinoma* OR melanoma* OR sarcoma*) AND TS=(innervat* OR
denervat* OR "nerve density" OR "nerve fiber*" OR "nerve fibre*" OR
"tumor-associated nerve*" OR "tumour-associated nerve*" OR "cancer
neuroscience" OR axonogenesis OR perineural OR sympathetic OR
parasympathetic OR "sensory nerve*" OR "sensory neuron*" OR "vagus
nerve" OR vagal OR autonomic OR nociceptor* OR "neural invasion") AND
TS=(NGF OR "nerve growth factor" OR BDNF OR GDNF OR artemin OR
neurturin OR TrkA OR TrkB OR NTRK* OR p75NTR OR semaphorin* OR
neuropilin* OR netrin* OR ephrin* OR SLIT2 OR ROBO1) AND TS=(mice OR
mouse OR murine OR rat OR rats OR rodent* OR xenograft* OR "in vivo" OR
"animal model*")
```

Module 4 — Safety net

Records returned: 4,318 · Export file: `WoS_Modul4_partea1…partea5_2026-08-23.ris`

```
TS=(cancer* OR tumor* OR tumour* OR neoplas* OR carcinoma* OR
adenocarcinoma* OR melanoma* OR sarcoma*) AND TS=("tumor innervation"
OR "tumour innervation" OR "tumoral innervation" OR "tumor-associated
nerve*" OR "tumour-associated nerve*" OR "intratumoral nerve*" OR
"intratumoural nerve*" OR "nerve density" OR "cancer neuroscience" OR
"neuro-neoplastic" OR "nerve-tumor*" OR "nerve-tumour*" OR "neuron-
cancer" OR "cancer-nerve" OR "neuron-tumor*" OR axonogenesis OR
neoneurogenesis OR "neo-neurogenesis" OR "perineural niche" OR "neural
invasion" OR "perineural invasion" OR innervat* OR denervat* OR
"sensory neuron*" OR nociceptor* OR "sympathetic nerve*" OR
"sympathetic innervation" OR "parasympathetic innervation" OR "vagal
innervation" OR "neural activity" OR glutamatergic OR neuroimmune OR
"neuro-immune") AND TS=(mice OR mouse OR murine OR rat OR rats OR
rodent* OR xenograft* OR "in vivo" OR "animal model*")
```

Sentinel query

Short query run before each module, to verify that the syntax had been transferred correctly into the interface. It must return the article Zhao CM et al., *Denervation suppresses gastric tumorigenesis*, Sci Transl Med 2014 (PMID 25143365).

```
"Denervation suppresses gastric tumorigenesis", Sci Transl Med 2014.
TS=(denervation AND gastric AND tumorigenesis)
```

S2.8. Supplementary searches

Reference-list checking. The reference lists of all included articles and of the major reviews in the field were checked manually.

Set of known references. The relevance filter applied after deduplication was validated on a set of 113 references independently identified as relevant before the search. None was lost by the filter.

Preprints. bioRxiv and medRxiv were consulted for the sensitivity analysis. The deduplication rule for multiple reports (amendment 6) required retaining a single version per study; in one case, a bioRxiv preprint and the published version in *Neuron* turned out to be the same study and were merged.

S2.9. Deduplication

Deduplication was not done in standard bibliographic software, but through a two-step procedure, reproducible as follows:

1. **Normalised DOI —** removal of resolver prefixes, conversion to lowercase, removal of spaces. Exact match.
2. **Normalised title —** removal of diacritics, punctuation and multiple spaces, conversion to lowercase, with a one-year tolerance for the publication year, to catch records where one database indexes the print-edition year and another the online-publication year.

From 33,432 gross records, 16,649 unique records resulted.
