# Supplementary material for: Molecular Targeting of the Peripheral Nerve–Tumor Axis in Extracranial Solid Cancers: Eight Signaling Systems, One Effect—Systematic Review and Multilevel Meta-Analysis
Source: Int J Mol Sci. 2026 Oct 14;27(20):9090. doi: 10.3390/ijms27209090 (PMC13705328; doi:10.3390/ijms27209090)
Supplement: Supplementary file 1 [file ijms-27-09090-s001.zip › S3_Data_extraction_form_and_controlled_vocabular.pdf]

Supplementary Material S3. Data extraction form and controlled vocabulary

Meta-analysis of the peripheral nerve–tumour axis in extracranial solid cancers

This document reproduces the form applied during data extraction, the operational definitions of each field, and the values actually observed in the final dataset. The form was piloted on 20 articles before extraction proper began.

S3.1. The unit of extraction: article → experiment → effect

The dataset has three nested levels, which correspond to the three levels of the statistical model.

Level	Definition	Number in the final dataset
**Article**	an eligible publication	363 (of which 352 with extractable full text)
**Experiment**	a unique combination of *tumour model × intervention*	1,295 (of which 1,274 with at least one effect)
**Effect**	an intervention-versus-control comparison for one outcome	1,441

The rules governing the split:

- The same tumour model tested with two different interventions = **two experiments**.
- The same intervention tested in three tumour models = **three experiments**.
- Multiple doses of the same intervention = **one effect per dose**, with `control_comun = Da`.
- Multiple measurement time points = **a single effect**, at the last time point common to all groups.
- Multiple outcomes = **a single effect**, chosen according to the hierarchy in S3.2.

S3.2. Primary outcome hierarchy

A level is stepped down to only if the level above is missing from the article.

Level	Outcome	Typical units
1	Tumour mass at sacrifice	mg, g
2	Tumour volume at the last common time point	mm^3

Level	Outcome	Typical units
3	Histological tumour area	mm^2, % of field
4	Bioluminescent or imaging signal	photons/s, radiance
5	Extent of perineural invasion, measured **continuously**	mm, mm^3, numeric score

Level 5 was added through **amendment 1** (S1), after extraction and before analysis.

Not accepted as an outcome: binary incidence of perineural invasion; sciatic nerve function score; intratumoural innervation density; count of invaded nerves per field. Effects that measured only such quantities were recorded in the database but flagged as outside the hierarchy and excluded from the model.

Hierarchy level	Effects extracted
1 — tumour mass	456
2 — tumour volume	494
3 — histological area	138
4 — bioluminescent signal	122
5 — continuous perineural invasion	91
6 — linear diameter (outside the hierarchy)	42
9 — other outcome (outside the hierarchy)	98

S3.3. Numeric fields and extraction rules

Field	Definition	Rule
`n_int`, `n_ctrl`	number of **animals**, as independent experimental units	not the number of tumours, sections or microscopic fields; if given as a range (“n = 5–7”), the minimum value is taken and noted in the observations
`media_int`, `media_ctrl`	arithmetic group mean	in the unit reported by the article
`variabilitate_int`, `variabilitate_ctrl`	reported dispersion	the raw value, **without conversion at extraction**
`tip_variabilitate`	what the value above actually is	`SEM` / `SD` / `IQR` / `min-max` / `not_reported`
`control_comun`	the control group is shared with another effect from the same experiment	`Yes` / `No`
`sursa_date`	where the value comes from	`text` / `table` / `supplementary_material` / `figure`

Field	Definition	Rule
`figura_de_digitizat`	the exact figure and panel	mandatory when `sursa_date = figure`

Order of sources at extraction: text → tables → supplementary material → figures.

Dispersion conversion was not done at extraction but at analysis, to preserve traceability: SD = SEM × √n; SD ≈ IQR / 1.35; SD ≈ range / 4. When the type was not declared, SEM was assumed, the dominant convention in the field, and the assumption was tested through a sensitivity analysis.

Per-animal individual values, when the article published them in a source data file, were preferred: the mean and standard deviation were recalculated from the raw data, and the sample size came out exact. Four of the 31 articles checked did publish such files.

S3.4. Moderators — controlled vocabulary and observed values

The tables below give, for each moderator, the values allowed by the protocol and the number of experiments (or articles) that actually received them in the final dataset.

The count refers to the **defined experiments** — all 1,295 — including the 21 that ultimately provided no usable effect. The experiments that actually contribute at least one effect number 1,274.

`nerv` — the targeted nerve fibre

The fibre whose modification was **demonstrated**, not the one presumed from the intervention's theoretical mechanism. When the article does not document which fibre was affected, or when the intervention reaches several, it is coded `Mixed-unspecified`.

Value	Experiments
Sympathetic	437
Mixed-unspecified	392
Sensory	339
Parasympathetic	126
Parasympathetic	1

`nivel` — molecular level of the intervention

The six levels set out in the protocol, plus the activation category introduced through **amendment 5**.

Value	Experiments
Activation-stimulation of the axis	301

Value	Experiments
Structural denervation	288
Neurotransmitter-receptor blockade	204
Anti-neurotrophin-anti-axonogenesis	180
Neuro-immune modulation	102
Functional silencing	97
Neuropeptide blockade	88
Other	35

`moment` — timing of the intervention relative to tumour stage

The central moderator of the review. **T0** = the intervention precedes inoculation or induction. **T1** = after inoculation, before the tumour is measurable. **T2** = after the tumour has become measurable or was confirmed. **T3** = after documented metastasis or progression. When the article does not allow the timing to be established, it is coded `Not reported` — it is not guessed.

Value	Experiments
T0	615
T1	299
T2	270
Not reported	106
T3	5

`engagement` — neural target engagement

Confirmed = the article actually measures nerve density, neuronal activity, or local neurotransmitter concentration after the intervention. **Probable** = engagement is supported indirectly, for example through method validation in another experiment of the same article. **Not confirmed** = no verification.

Value	Experiments
Confirmed	593
Probable	514
Not confirmed	188

`administrare` — route of administration

The `Genetic` category covers knockout, knock-in and conditional models, in which “administration” is constitutive or genetically induced.

Value	Experiments
Systemic	701

Value	Experiments
Local	348
Genetic	246

`model` — tumour model

The experimental model through which the tumour was obtained.

Value	Experiments
Orthotopic	459
Subcutaneous-syngeneic	172
Perineural invasion-sciatic nerve	125
GEMM	122
Subcutaneous-xenograft	119
Orthotopic-GEMM	104
Other	104
Metastatic-i.v. injection	31
PDX	13
Orthotopic-GEMM (syngeneic model of pulmonary colonisation after i.v. injection)	11
Metastatic-intrasplenic injection	11
Orthotopic-GEMM (orthotopic syngeneic allograft — intravesical instillation)	4
Orthotopic-GEMM (syngeneic pulmonary transplant, i.v.)	4
Orthotopic-GEMM (orthotopic syngeneic allograft in the pancreas)	3
Orthotopic-GEMM (orthotopic syngeneic allograft — intravesical instillation) in Rag2-KO hosts	1
Subcutaneous-xenograft (subcutaneous syngeneic allograft, in the nape of the neck)	1
Orthotopic-GEMM (syngeneic pulmonary transplant via i.v. injection into the tail vein — 'orthotopic transplantation model' in the authors' terminology)	1
Orthotopic-GEMM (syngeneic pulmonary transplant, i.v.), with prior exposure to cigarette smoke extract (CSE)	1
Orthotopic-GEMM (syngeneic pulmonary transplant, i.v.), animals not exposed to CSE (vehicle)	1
Orthotopic-GEMM (syngeneic pulmonary transplant, i.v.), animals exposed to CSE	1
Subcutaneous-xenograft (subcutaneous syngeneic allograft in an immunocompetent mouse)	1

Value	Experiments
Orthotopic-GEMM (neural invasion model: tumour cells implanted at the periphery of the sciatic nerve)	1
Subcutaneous-xenograft (subcutaneous syngeneic allograft in the hind limb; a non-orthotopic site chosen explicitly for repeated calliper measurement)	1
Subcutaneous-xenograft (subcutaneous syngeneic allograft in the hind limb), with associated chemotherapy	1
Orthotopic-GEMM (syngeneic model of pulmonary colonisation after i.v. injection; does not fit strictly into either category)	1
Subcutaneous-xenograft (in fact a subcutaneous syngeneic allograft, popliteal fossa)	1
Other: experimental pulmonary metastasis (i.v. injection into the tail vein)	1

`tumora` — tumour location

The location was recorded at extraction as free text and normalised at analysis into a closed vocabulary; both forms are kept in the database (S4).

Value	Experiments
Pancreas	260
Breast	244
Colon-rectum	110
Stomach	101
Lung	98
Prostate	93
Skin-melanoma	92
Oral cavity	78
Other	65
Head-and-neck	41
Ovary	40
Liver	37
Bone-sarcoma	20
Bladder	7
Oesophagus	5
Kidney	4

Article-level fields

`specie` — Animal species.

Value	Articles
mouse	329
rat	17
Mouse	8
not reported in the abstract (very probably mouse)	2
mouse (per the abstract; details unavailable)	2
other	2
Not reported in the abstract	1
mouse (probable — 'in vivo' unspecified in the abstract)	1
Mouse (BALB/c nude mice; Sprague-Dawley rats used ONLY on	1

`sex` — Sex of the animals.

Value	Articles
male	106
female	88
not reported	88
male and female	35
not reported in the abstract	4
Not reported	4
	3
Female	3
Not reported in the abstract	2
females (6–12 weeks at implantation; RTX administered at 4 weeks	1
both sexes, aged 8–12 weeks, with sex-stratified randomisation	1
females	1

`imunitate` — Immune status of the host.

Value	Articles
Immunocompetent	219
Immunodeficient	130
not reported in the abstract	4
	3
Not reported in the abstract	2
Immunocompetent for most experiments (C57BL/6J and transgen	1
Immunodeficient (BALB/c-nude, exp. 1) and Immunocompetent (C57BL/6J, exp.	1
Immunocompetent (EO771 in C57BL/6 and TRPV1-ChR2);	1

Value	Articles
Immunodeficient (PDX i	
Immunocompetent (syngeneic models)	1
Immunocompetent (syngeneic allograft on a C57BL/6 background)	1

`randomizare` — The article explicitly declares randomisation of allocation.

Value	Articles
No-not reported	214
Yes	139
	3
Yes — 'Mice were randomly assigned to all the experimental groups. Rand	1
Yes — 'Post-inoculation, the cohort were randomized into four distinct	1
No-not reported (allocation is determined by genotype: Nes-cre+ vs Nes-c	1
Yes (the mice were 'randomly divided'/'randomly assigned' into groups	1
Yes (stated: 'Study groups were balanced with males and females in ea	1
No-not reported (sample size set through a power analy	1
No-not reported (group allocation is determined by genotype: Cre+ v	1

S3.5. Risk of bias — SYRCLE

The ten SYRCLE domains, assessed for every eligible article. Values: **Low** / **Unclear** / **High**.

Code	Domain	Type of bias
D1	Sequence generation	Selection bias
D2	Similar baseline characteristics	Selection bias
D3	Allocation concealment	Selection bias
D4	Random housing of animals	Performance bias
D5	Blinding of caregivers and investigators	Performance bias
D6	Random selection for outcome assessment	Detection bias
D7	Blinding of the outcome assessor	Detection bias
D8	Incomplete outcome data	Attrition bias
D9	Selective outcome reporting	Reporting bias
D10	Other sources of bias	Other bias

The coding convention, applied identically to all articles: **"Unclear" means the article is silent** on the domain, not that the procedure was incorrect. **"High" means the article describes a procedure that introduces bias**, or explicitly declares it absent.

Domain D10 was used predominantly for **pseudoreplication**: reporting histological sections, microscopic fields, or tumours — rather than animals — as the experimental unit. The full article-by-article assessment is in S6.

S3.6. Standing instructions added during extraction

Three rules were added to the form after extraction had begun, in response to concrete problems. They were applied retroactively to all articles already extracted.

1. **Visual check of the first page**, for retraction notices. One retracted article carried the notice only as a watermark image, invisible both to PubMed indexing and to the text extracted from the PDF.
2. **MD5 fingerprinting of every PDF**, to detect files downloaded twice under different numbers. The procedure identified four such pairs.
3. **Title matching restricted to the first ~2,500 characters** of the extracted text, to avoid false matches with titles appearing in another article's reference list.

S3.7. Digitisation of graphical panels

The full procedure is described in the Methods section of the manuscript. In brief: pages were rendered at 200 dpi, with crops at 600 dpi where the error bar was not legible; for each panel the extreme values and the gradations of the y-axis and the scale type — linear, logarithmic or broken — were identified, and values were obtained through the corresponding interpolation.

Each digitised value was given, at the time it was read, a confidence score:

Score	Criterion	Effects
`high`	clear axis, sharp bar ends	588
`medium`	interpolation between sparse gradations, broken scale, partially obscured bar end	331
`low`	dispersion estimated from individual points, logarithmic axis with no intermediate gradations, cluttered panel	109
`directly_reported`	value taken directly from text, a table, or a source data file	203

The score is kept as a variable in the database (S4) and used in a prespecified sensitivity analysis, reported in section 3.8 of the manuscript.
