# Supplementary material for: Molecular Targeting of the Peripheral Nerve–Tumor Axis in Extracranial Solid Cancers: Eight Signaling Systems, One Effect—Systematic Review and Multilevel Meta-Analysis
Source: Int J Mol Sci. 2026 Oct 14;27(20):9090. doi: 10.3390/ijms27209090 (PMC13705328; doi:10.3390/ijms27209090)
Supplement: Supplementary file 1 [file ijms-27-09090-s001.zip › S8_Screening_reliability.pdf]

Supplementary Material S8. Screening reliability

Meta-analysis of the peripheral nerve–tumor axis in extracranial solid cancers

---

S8.1. Procedure

Title and abstract screening was carried out in two independent passes over the same dataset of 10,731 abstracts, using the same criteria form (reproduced in S8.5).

Pass 1 classified each record into three categories — *Include*, *Uncertain*, *Exclude* — with the explicit instruction to use the *Uncertain* category generously, to protect sensitivity at the expense of precision. Any *Include* or *Uncertain* record was sent forward to full-text assessment, so that an *Uncertain* classification excluded no article.

Pass 2 independently re-assessed a random sample of 240 records, stratified across the three categories of the first pass, with no access to the initial decision and no access to its reason. The sample deliberately included 80 records classified *Exclude* at the first pass — the recovery check that PRISMA requires, i.e. the test of whether screening had lost eligible studies.

Disagreements were not resolved algorithmically. They were extracted into a separate list and submitted for adjudication by the two authors, each with access to the title, abstract, and the reason recorded by the second pass (file `Validare_screening_PRIORITIZATA.xlsx`, sheet `DE_ARBITRAT`).

S8.2. Observed agreement

Indicator	Value
Records independently re-assessed	240
Raw agreement, across three categories	69.2%
Cohen's kappa, across three categories	**0.537**
Agreement on the binary decision (retained vs excluded)	75.0%
Kappa on the binary decision	**0.526**
Total disagreements	74
— of which change the article's fate	60

S8.3. Confusion matrix

Pass 1 in rows, pass 2 in columns.

Pass 1 ↓ / Pass 2 →	Include	Uncertain	Exclude	Total
**Include**	73	2	5	80

Pass 1 ↓ / Pass 2 →	Include	Uncertain	Exclude	Total
**Uncertain**	12	13	55	80
**Exclude**	0	0	80	80
**Total**	85	15	140	240

S8.4. Interpretation

A kappa of 0.54 falls in the moderate-agreement range. The raw figure, however, underestimates the operational reliability of the screening, for three reasons that can be read directly from the matrix.

First, and most important: the recovery check passed perfectly. Of the 80 records excluded at the first pass and independently re-assessed, **none** was reclassified as *Include* or *Uncertain* (row "Exclude": 0 / 0 / 80). The error that matters in a systematic review — losing an eligible study — did not occur in the sample checked.

Second: disagreement is concentrated in a single cell. 55 of the 74 disagreements are *Uncertain* → *Exclude* transitions, i.e. situations in which the second pass was more decisive than the first, not situations in which the two passes contradicted each other on substance. This is the direct consequence of the instruction to use the *Uncertain* category generously: it inflates apparent disagreement precisely because it moves the uncertainty out of the exclusion decision and into the intermediate category, where it is visible and recoverable.

Third: reversals in the dangerous direction are rare. Only 5 of the 80 records classified *Include* at the first pass were excluded at the second, and all five were submitted to human adjudication, not resolved automatically.

The limitation remains real. Reliability was estimated on 240 records, not on the entire dataset of 10,731. We cannot rule out that eligible studies were lost at the first pass among the 10,491 unverified records. The confidence interval for the loss rate, estimated on 80 verified exclusions with no failures, is 0–4.5% (rule of three, 95% CI).

S8.5. Criteria applied at screening

The criteria reproduced below are those used identically at both passes.

Retained are in vivo mammalian studies, on extracranial solid cancers, in which there is an intervention that modulates tumor innervation, peripheral nerve activity, or neural signaling, and at least one tumor-burden outcome.

Excluded are studies that are exclusively in vitro; studies in human subjects; reviews, commentaries and editorials; central nervous system tumors and tumors of the nerve itself; studies with no intervention on the axis; and studies with no tumor-burden outcome.

Marked *Uncertain* — and passed forward to full text — are records for which the abstract does not allow a decision, in particular those with no abstract, those in which the neural intervention is mentioned without it being clear whether it was applied in vivo, and those in which the tumor outcome is mentioned without specifying the measure.

S8.6. Associated files

File	Content
`Validare_screening_PRIORITIZATA.xlsx`, sheet `DE_ARBITRAT`	the 74 disagreements, with title, abstract, each pass's decision and the reason for the second one
same, sheet `ESANTION_COMPLET`	all 240 re-assessed records
same, sheet `Statistici`	the indicators from S8.2 and the matrix from S8.3

S8.7. Outcome of the adjudication

The 74 disagreements were adjudicated after the analyses reported in the manuscript had first been run, and the review was updated accordingly. The outcome was as follows.

Sixty-nine records were excluded. None of them had reached full text: 67 had been marked Uncertain at the first pass and 2 Include, and all 69 were among the reports that could not be retrieved. Excluding them therefore moves 69 records from "reports sought for retrieval" to "excluded at title/abstract" in the flow diagram, and changes no effect estimate, because these records contributed no extracted data.

Five records had reached full text and had been judged eligible. Four were retained. For each of these four, the reason recorded at the second screening pass was contradicted by the full text: one article marked as a review reports two in vivo experiments; one marked as having no neural intervention uses chronic restraint stress, which is an axis-activation intervention under this protocol; and one marked as having no tumor outcome does report one. The principle applied is that a full-text eligibility decision supersedes a title/abstract screening decision, since screening only determines what is read.

One article was excluded — an epigenetic manipulation of the tumor cell (KDM3B knockdown) with no intervention on the nerve. It contributed 4 effects to the model.

Effect on the results. The eligible set moved from 363 to 362 articles, and the model from 1,111 to 1,107 effects. The pooled estimate for axis blockade moved from −44.9% to −44.8% (95% CI: −48.3 to −40.9); the pre-specified primary analysis (−52.0%) and the activation arm (+52.1%) were unchanged. All figures, tables and supplementary files were regenerated on the updated dataset.
